# Efficient production of astaxanthin in *Yarrowia lipolytica* through metabolic and enzyme engineering

**DOI:** 10.1016/j.synbio.2025.02.014

**Published:** 2025-02-28

**Authors:** Chalak Najat Abdullah, Mengsu Liu, Qihang Chen, Song Gao, Changtai Zhang, Shike Liu, Jingwen Zhou

**Affiliations:** a School of Biotechnology, Jiangnan University, 1800 Lihu Road, Wuxi, Jiangsu, 214122, China; b Department of Biology, College of Science, University of Sulaimani, 46001, Sulaimaniyah, Kurdistan region, Iraq; c Engineering Research Center of Ministry of Education on Food Synthetic Biotechnology, Jiangnan University, 1800 Lihu Road, Wuxi, Jiangsu, 214122, China; d Science Center for Future Foods, Jiangnan University, 1800 Lihu Road, Wuxi, Jiangsu, 214122, China; e Jiangsu Provisional Research Center for Bioactive Product Processing Technology, Jiangnan University, 1800 Lihu Road, Wuxi, Jiangsu, 214122, China

**Keywords:** Astaxanthin, Modular enzyme assembly, Enzyme engineering, Subcellular organelles, *Yarrowia lipolytica*

## Abstract

Astaxanthin is a natural red carotenoid, commonly used as an additive in the pharmaceutical industry and as a nutritional supplement owing to its notable antioxidant benefits. However, a complex biosynthetic pathway poses a challenge to *de novo* biosynthesis of astaxanthin. Here, *Yarrowia lipolytica* was engineered through multiple strategies for high level production of astaxanthin using a cheap mineral medium. For the production of β-carotene, a platform strain was constructed in which 411.7 mg/L of β-carotene was produced at a shake-flask level. Integration of algal β-carotene ketolase and β-carotene hydroxylase led to the production of 12.3 mg/L of astaxanthin. Furthermore, construction of *HpBKT* and *HpCrtZ* as a single enzyme complex along with the enhanced catalytic activity of the enzymes led to the accumulation of 41.0 mg/L of astaxanthin. Iterative gene integration into the genome and direction of the astaxanthin production pathway into sub-organelles substantially increased astaxanthin production (172.1 mg/L). Finally, restoration of the auxotrophic markers and medium optimization further improved astaxanthin production to 237.3 mg/L. The aforementioned approaches were employed in fed-batch fermentation to produce 2820 mg/L of astaxanthin (229-fold improvement regarding the starter strain), with an average productivity of 434 mg/L/d and a yield of 5.6 mg/g glucose, which is the highest reported productivity in *Y. lipolytica*.

## Graphical abstract

**Figure:**
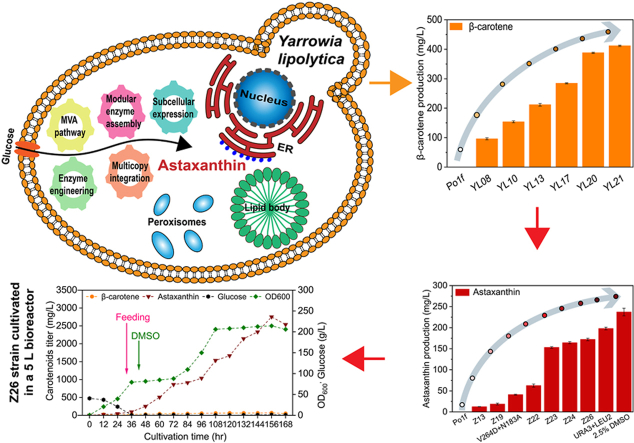

## 1 Introduction

As natural products, carotenoids exhibit a variety of colors ranging from red to yellow and occur as organic pigments in nature. Cyanobacteria, algae, and photosynthetic plants, as well non-photosynthetic fungi, yeast, and bacteria play a crucial role in the manufacture of different types of carotenoids, particularly astaxanthin, zeaxanthin, and lutein that have crucial roles as pigments. Astaxanthin is a bright red carotenoid containing keto groups and is well-known for its strong antioxidant effects. Specifically, astaxanthin has anticancer, anti-inflammatory, and antioxidant properties that make it suitable for use as nutraceuticals, fish or cattle feed, and cosmetics [[1], [2], [3]]. The worldwide consumer demand for astaxanthin is expected to have a valuation of $3.40 billion by 2027 [4].

Consequently, a variety of approaches for producing astaxanthin have been investigated. These approaches include chemical synthesis, cultivation of natural producers, such as *Paracoccus* sp. (bacteria), *Xanthophyllomyces dendrorhous* (yeast), *Haematococcus pluvialis* (microalgae), and direct extraction from crustacean waste [[5], [6], [7]]. Astaxanthin is present in two enantiomeric forms, namely (3*R*,3′*R*) and (3*S*,3′*S*), as well as in a meso form designated as (3*S*,3′*R*), owing to the presence of two stereogenic carbon atoms located at the C3 and C3′ positions [8]. The yeast *X. dendrorhous* accumulates 3*R*,3′*R*-astaxanthin at 2 % of dry cell weight [9], whereas *H. pluvialis* mostly produces the (3*S*,3′*S*) isomer of astaxanthin. In addition, chemically produced astaxanthin has a stereoisomeric ratio of 1:2:1 for the (3*S*,3′*S*), (3*S*,3′*R*), and (3*R*,3′*R*) isomers [10]. Among bacteria and yeast cells which have been widely used to manufacture of natural compounds, *Escherichia coli* [11], *Saccharomyces cerevisiae* [12], *X. dendrorhous* [13], and *Yarrowia lipolytica* [14] have been identified as candidate organisms that can initiate the astaxanthin biosynthetic pathway. Astaxanthin is synthesized from a precursor, β-carotene that requires two enzymes, namely β-carotene ketolase (*CrtW*) [15] and β-carotene hydroxylase (*CrtZ*) [16]. These enzymes produce eight intermediate molecules, including astaxanthin, by catalyzing four convertible processes that start with β-carotene. *CrtZ* is an enzyme with two functions. The enzyme catalyzes hydroxylation of β-carotene to produce zeaxanthin via the conversion of β-cryptoxanthin. The enzyme also converts canthaxanthin into zeaxanthin via adonirubin [17]. *Escherichia coli* produced a maximum titer of astaxanthin of 1.82 g/L [3]. Additionally, *Saccharomyces cerevisiae* (446.4 mg/L) [18] and *Yarrowia lipolytica* (54.6 mg/L–3.3 g/L) have produced substantial amounts of astaxanthin [19,20].

*Y. lipolytica* has numerous benefits over *S. cerevisiae* in the biosynthesis of astaxanthin, including ability to spontaneously create substantial quantities of cytosolic acetyl-CoA which is the precursor for astaxanthin production [21,22], and availability of several genetic approaches to systematically generate *Y. lipolytica* strains [23,24]. Additionally, *Y. lipolytica* can rely on a broad range of carbon-containing substances including waste oils, molasses [25] and glycerol [26], which potentially promoting sustainable biological manufacturing. The preliminary accomplished astaxanthin synthesis in the engineered *Y. lipolytica* yielded a titer level of 54.6 mg/L (3.5 mg/g dry cell weight), which was achieved by overexpressing native genes involved in the mevalonate (MVA) pathway and incorporating exogenous genes associated with β-carotene production from *X. dendrorhous*, as well as regulation of squalene synthase expression and fine-tuning gene copy numbers of β-carotene hydroxylase (*CrtZ*) and β-carotene ketolase (*CrtW*) [19]. A unique technique has been described by Kang et al. which involves fusion of each two enzymes with regular interaction-inducing double helix (RIDD) and regular interaction-inducing amphiphilic double helix (RIAD) peptides to decrease intermediates diffusion in the synthetic pathways [27]. Astaxanthin titer of 3.3 g/L (26.5 mg/g DCW) has been produced in *Y. lipolytica* during 11 days of fermentation in a 5-L bioreactor through multicopy integration of fused *HpBKT* and *HpCrtZ* [20]. However, the approach demands a consistent provision of costly and high-quality media, which renders it unfeasible for commercial use.

At the moment, build-up of intermediates and limited catalytic activity of ketolases and hydroxylases are major obstacles in the astaxanthin synthesis by microbial chassis. Therefore, in this study *Y. lipolytica* was systematically engineered for the efficient and high-level synthesis of 3*S*,3′*S* astaxanthin through employing multiple strategies, including semi-rationally design of β-carotene hydroxylase from *H. pluvialis* for the first time. Initially, 411.7 mg/L of β-carotene was achieved through construction of the β-carotene synthetic pathway and optimizing the MVA pathway. By screening β-carotene ketolases and β-carotene hydroxylases from various sources, astaxanthin was successfully produced in the engineered strains. Construction of β-carotene ketolase and β-carotene hydroxylase as a single enzyme complex via applying modular enzyme assembly considerably decreased intermediates diffusion in the synthetic pathway and enhanced accumulation of the final product. Astaxanthin production was further boosted through increasing catalytic activity of the enzymes in the astaxanthin synthetic pathway, iterative integration of engineered enzymes, and subcellular compartmentalization. Finally, fine-tuning of fed-batch fermentation using a cheap inorganic salt medium for the first time increased astaxanthin yield to 2820 mg/L, which shows substantial benefits of *Y. lipolytica* for industrial scale production of astaxanthin.

## 2 Materials and methods

### 2.1 Strains and medium

To propagate plasmids, *Escherichia coli* JM109 cells were cultured in Luria–Bertani (LB) medium supplemented with 100 mg/mL ampicillin at 37 °C for 16 h. Competent *E. coli* cells were generated using a competent cell kit (TaKaRa Bio Inc., Osaka, Japan). The basic host strain employed was *Y. lipolytica* Po1f (ATCC-MYA 2613) [28]. All *Y. lipolytica* strains were incubated at 30 °C and 220 rpm on yeast extract peptone dextrose (YPD) medium (g/L) consisting of yeast extract 10, peptone 20, and glucose 20 to activate the strain or on yeast nitrogen base (YNB) medium (g/L) consisting of 6.74 YNB, glucose 20, and leucine or uracil (5 g/L) to support auxotrophic growth and facilitate selection of altered *Y. lipolytica* strains.

### 2.2 Plasmids construction

All plasmids and heterologous genes used in this study to investigate β-carotene and astaxanthin synthesis are listed in Tables S1 and S2, respectively. All heterologous genes were codon optimized and synthesized by GENEWIZ Biotech Co., Ltd. (Suzhou, China). All the endogenous genes were amplified from *Y. lipolytica* Po1f genome using 2 × Phanta Max Master Mix (Dye Plus; Vazyme, Nanjing, China). To create plasmids for genome alteration, a set of YaliBricks vectors (Biovector NTCC Co., Ltd., Beijing, China) and MnDH2 (P_MnDH2_), TDH (P_TDH_) or TEF (P_TEF_) promoters, and the ICL (T_ICL_) or XPR2 (T_XPR2_) terminators were used to flank both endogenous and heterologous genes [29]. Sequences of the promoters and terminators were obtained from the genome of *Y. lipolytica* [28,29]. Plasmid fragments were constructed using the plasmid pYLXP'1 as a template with primer pair CarRP-F/R and CarB-F/R. After gel recovery, fragments of the plasmid were assembled using Gibson assembly kit (Sangon Biotech Co., Ltd., Shanghai, China) and transferred into *E. coli* JM109 competent cells, yielding plasmid pYL01. All the other vectors were constructed using distinct primer pairs synthesized by Sangon Biotech Co., Ltd. (Shanghai, China), in accordance with the above-described method. A set of primers (forward and reverse primer) with a specific point mutation were designed to introduce the specific point mutations into *HpcrtZ* and *HpBKT* original plasmids through performing PCR. All primers used in this study are listed in Table S3. All generated plasmids were verified using Sanger sequencing by Sangon Biotech Co., Ltd. (Shanghai, China).

### 2.3 Strains construction and yeast transformation

All strains constructed in this study are listed in Table S4. The genes necessary for β-carotene and astaxanthin production were incorporated into the genome of *Y. lipolytica* by homologous recombination. Integrated fragments were designed using the primer combination E2:*CarRP*^Y27R^-*CarB*−FL/RL, with the plasmid pYL01 acting as a template. For the integration of a specific gene, the homology donor plasmid and the CRISPR-Cas9 expression plasmid (containing the relevant guide RNA sequence and leucine marker) were introduced into the *Y. lipolytica* strain [30]. The guide RNAs were designed using an online CRISPR gRNAs tool (https://chopchop.cbu.uib.no/). The integrating sites used in this study were not vital genes in the genome of *Y. lipolytica*, as previously reported [31]. The *Y. lipolytica* transformation was conducted in accordance with the specifications of the Frozen-EZ Yeast Transformation II kit (Zymol Research, CA, USA). YNB medium agar plates were used to cultivate transformants at 30 °C for 2–4 days. To eliminate LEU2 marker, cells were cultured in YPD medium for 48 h. For multicopy integration at 26S recombinant DNA (rDNA) loci, the positive transformants were cultured on the uracil dropout YNB plate and the URA3 marker was cured as previously described [32].

### 2.4 Shake flask and bioreactor fermentation for carotenoid synthesis

To evaluate the ability of the engineered strains for β-carotene synthesis, fermentations was performed in 250 mL shaking flasks. Isolated colonies obtained from YPD plates after 48 h of incubation were randomly selected, transferred into 24 deep well plates containing 3 mL of YPD medium, and cultivated at 30 °C and 220 rpm with constant shaking for 20 h. Each 24 mL of the YPD liquid medium in 250 mL shaking flasks was inoculated with 1 mL of the broth culture and subsequently cultured at 30 °C at 220 rpm for 120 h. Astaxanthin producing strains were prepared using the same procedure. Afterward, 200 μL of the seed liquid was placed into 24 deep well plates containing 4 mL of YPD and fermentation was allowed to proceed under the same conditions.

To expand astaxanthin production, the strain engineered for astaxanthin synthesis was cultured through fed-batch fermentation in a 5 L bioreactor. The main culture solution was established by introducing five to seven isolated colonies into 10 mL YNB medium and cultured at 30 °C and 220 rpm for 24 h. Subsequently, the primary culture solution was transferred into 200 mL YNB medium. The mixture was then cultured for 24 h, leading to the preparation of the secondary culture solution. Finally, the secondary culture solution was introduced into a 5 L bioreactor containing 2200 mL of inorganic salt medium (7.5 g/L (NH4)_2_SO_4_, 3.5 g/L KH_2_PO_4_, 0.5 g/L MgSO_4_·7H_2_O, 40 g/L d-glucose, 10 mL vitamin solution and 12 mL trace metal solution) [33], and fed-batch fermentation was conducted under the following conditions: fermentation temperature of 28 °C, ammonia solution was supplied during fermentation to maintain pH at 5.5, and an agitation cascade ranging from 300 to 900 rpm was used to regulate the dissolved oxygen at 20 % when the air flow rate was 3.0 vvm. The fed-batch process started with (100 g/L (NH4)_2_SO_4_, 35 g/L KH_2_PO_4_, 5 g/L MgSO_4_·7H_2_O) being fed at a rate of 8 mL/h after the initial glucose was depleted. Glucose levels were consistently maintained below 1 g/L by supplying 500 g/L d-glucose to the bioreactor throughout the fermentation process.

### 2.5 Analytical methods

After five days of fermentation, three replicate samples were used to detect β-carotene, lycopene, and astaxanthin titers. Briefly, 100 μL of the fermentation broth was centrifuged at 10,000 g for 5 min, then, the liquid upper layer was removed and the collected cell pellets were resuspended in 0.75 mL dimethyl sulfoxide (DMSO) and incubated at 55 °C for 15 min. Acetone (0.75 mL) was added to the collected samples and incubated at 45 °C for 20 min. The samples were subsequently centrifuged at 10,000 g for 5 min. The resulting supernatant was filtered and quantified using a SHIMADZU LC-20AT high-performance liquid chromatography (HPLC) system equipped with a C18 column (250 nm × 4.6 mm, 5 μm; Thermo Fisher Scientific, Waltham, MA, USA). The mobile phase comprised acetonitrile-methanol-isopropanol (5:3:2 v/v/v). Carotenoids were detected at a flow rate of 1 mL/min, wavelength of 450 nm, and the column temperature was set to 40 °C. The standard curves for β-carotene, lycopene, and astaxanthin were constructed using commercial standards with the same method used for sample extraction. Astaxanthin verification was conducted using a Waters MALDI SYNAPT Q-TOF MS system (Waters, MA, USA) employing previously established methodologies in electrospray ionization (ESI) negative mode [33]. LC-Q-TOF/MS mass spectra of the astaxanthin standard and sample are presented in Fig. S6. Chiral HPLC analysis was performed as previously described (Fig. S9) [8].

To quantify the dry cell weight (DCW), 5 mL of the fermentation samples were centrifuged at 10,000 g for 10 min and the top liquid layer was eliminated. The collected cells were oven-dried at 65 °C for 48 h to achieve a consistent weight.

### 2.6 Direction of metabolic pathways to compartments within cells

Specific localization signals were used to target *HpBKT-HpCrtZ* to various compartments inside the engineered strains (i.e., the endoplasmic reticulum [ER], lipid bodies, and peroxisomes), as previously described [34]. The enzymes were directed to the ER by adding KDEL with the oligonucleotide sequence 5′-AAGGACGAGCTG-3′ at the C-terminus and eliminating the stop codon to the end of targeting signal. SKL or oleosin from *Zea mays* was added to ensure protein targeting in peroxisomes or lipid bodies. Sequence of oleosin with codon-optimized for *Y. lipolytica* is listed in Table S2. Laser scanning confocal microscope (LSCM) was used to examine the dispersion of red fluorescent protein (mCherry) to assess the targeting efficacy of the addressing signals to target ER, lipid bodies, and peroxisome (Fig. S7).

## 3 Results

### 3.1 Engineering of the β-carotene biosynthetic pathway in *Yarrowia lipolytica*

**Fig. 1:**
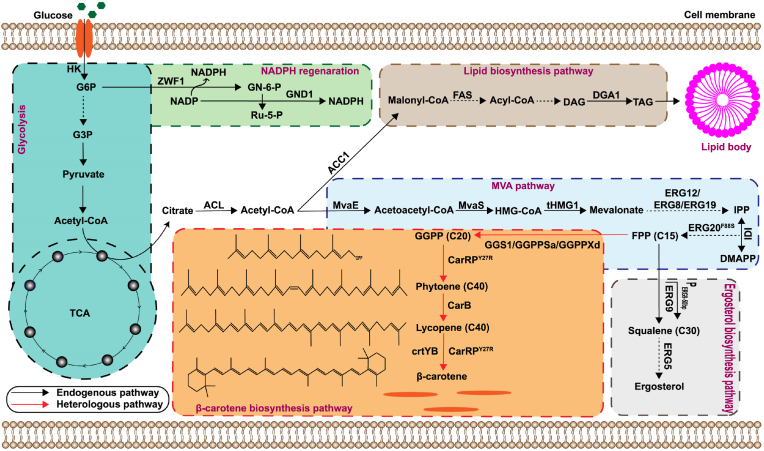
A schematic illustration of an engineered strain designed for β-carotene synthesis. G6P, Glucose 6-phosphate; G3P, glyceraldehyde-3-phosphate; *Zwf1*, glucose-6-phosphate dehydrogenase; *Gnd1*, 6-phosphogluconate dehydrogenase; *Acc1*, acetyl-CoA carboxylase; *Dga1*, acyl-CoA diacylglycerol acyltransferase; TAG, triacylglycerol; *MvaE*, acetyl-CoA acetyltransferase/HMG-CoA reductase; *MvaS*, HMG-CoA synthase; *tHmg1*, truncated HMG1; *Erg9*, squalene synthase; *Erg12*, mevalonate kinase; *IPP*, isopentenyl pyrophosphate; DMAPP, dimethylallyl pyrophosphate; FPP, farnesyl pyrophosphate; GGPP, geranylgeranyl pyrophosphate; *IDI*, IPP isomerase; *Erg20*, geranyl/farnesyl diphosphate synthase; *GGS1*/*GGPPXd*/*GGPPSa*, GGPP synthase; *CarRP* and *CrtYB*, phytoene synthase/lycopene cyclase; *CarB*, phytoene dehydrogenase.

**Fig. 2:**
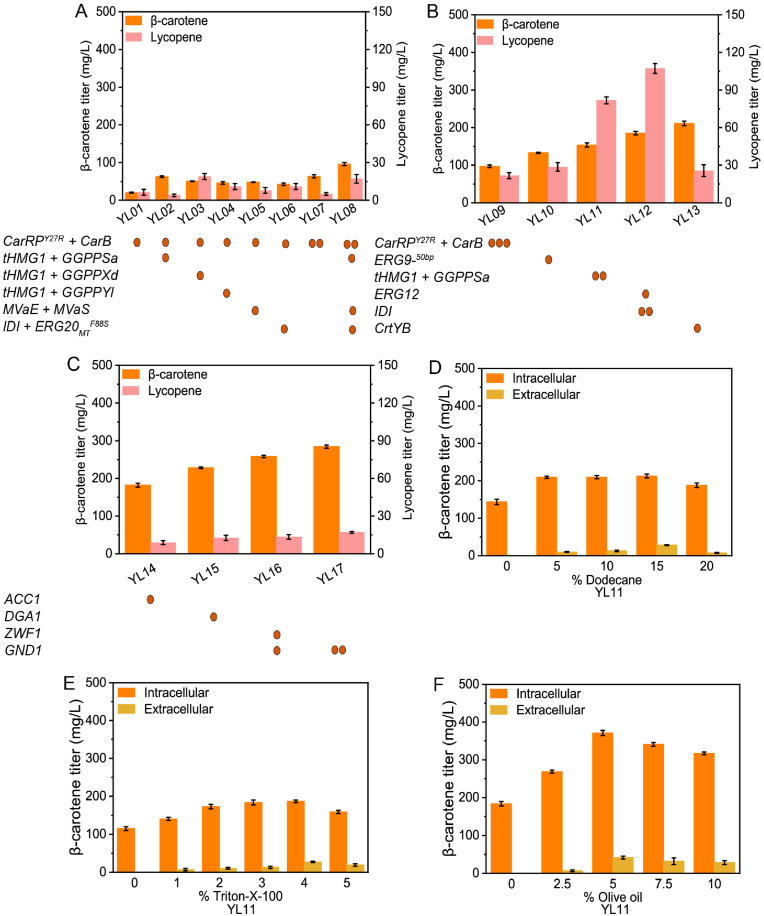
Construction of strains capable of producing carotenoids. (A) Construction of β-carotene biosynthetic pathway, Screening GGPP synthase from different sources, and overexpression of essential genes in the MVA pathway. (B) Increasing copy numbers of essential genes in the MVA and β-carotene biosynthetic pathways together with downregulation of squalene competitive pathway. (C) Upregulation of the expression levels of genes involved in the production of fatty acids and enhancement of NADPH supply in the β-carotene-producing strain (D) β-carotene titer in strain YL11 following treatment with various doses of dodecane. (E) β-carotene titer in strain YL11 following treatment with various doses of Triton-X-100. (F) β-carotene titer in strain YL11 following treatment with various doses of olive oil. "" indicates the genes has been incorporated into the chromosome of the specific strain. The bar heights represent the mean of three independent experiments and the error bars represent the standard deviations.

Multiple enzymes are involved in β-carotene production from acetyl-CoA through a long metabolic pathway (Fig. 1). To facilitate heterologous β-carotene synthesis in *Y. lipolytica* Po1f, we initially introduced fundamental genes for β-carotene synthesis, namely *CarRP*^Y27R^ and *CarB* from *Mucor circinelloides* into the *Y. lipolytica* genome. The transformed strain YL01 attained a β-carotene level of 20.3 mg/L (Fig. 2A). *HMG1* and *GGPPS* are key genes associated with the MVA pathway. Our approach initiated with overexpression of the truncated version of *HMG1* (*tHMG1*) and screening of GGPP synthase from different sources such as, *GGPPSa* from *Sulfolobus acidocaldarius*, *GGPPxd* from *X. dendrorhous*, and endogenous *GGPPYl* from *Y. lipolytica* into YL01 to construct YL02-YL04. Among the screened enzymes, overexpression of *tHMG1* with *GGPPSa* from *Sulfolobus acidocaldarius* produced the highest amount of β-carotene (62.6 mg/L) in YL02 (Fig. 2A).

Enhanced synthesis of β-carotene from MVA led to MVA depletion. Therefore, foreign genes, *MvaE* and *MvaS* from *Enterococcus faecalis* were introduced into YL01 to develop YL05, which yielded β-carotene titer of 48.2 mg/L (Fig. 2A). To further improve the MVA pathway, *IDI* and *Erg20*_MT_^F88S^ were overexpressed in YL01, which yielded β-carotene titer of up to 42.6 mg/L in YL06 (Fig. 2A). The introduction of another copy of *CarRP*^Y27R^ and *CarB* in YL01, yielded a β-carotene titer of 65.3 mg/L in YL07 (Fig. 2A). The introduction and overexpression of all the aforementioned genes into YL01 to obtain YL08 led to β-carotene and lycopene titers of 96.0 and 17.1 mg/L, respectively (Fig. 2A). However, a further increase in the copy numbers of *CarRP*^Y27R^ and *CarB* to three copies in YL08 did not improve β-carotene synthesis (YL09, Fig. 2B).

To provide more farnesyl pyrophosphate for carotenoid biosynthesis, we decreased the flux toward squalene production in strain YL08 by truncating the native promoter of squalene synthase to 50 base pairs. The transformed strain YL10 exhibited an increase in β-carotene titer (133 mg/L) and accumulated 28.6 mg/L of lycopene (Fig. 2B). The introduction of another copy of *tHMG1*and *GGPPSa* into strain YL10 yielded a β-carotene titer of up to 153.8 mg/L, as well as a precursor substance, lycopene, at a concentration of 81.8 mg/L (Fig. 2B) in strain YL11. Overexpression of *Erg12* with another copy of *IDI* in YL11 led to a β-carotene titer of 185.4 mg/L in strain YL12, whereas lycopene accumulated to a concentration of 107.2 mg/L (Fig. 2B).

To promote the conversion of the accumulated lycopene in the engineered strain, we simultaneously introduced lycopene cyclase *CrtYB* from *X. dendrorhous* in the strain YL12, which led to a decrease in lycopene concentration (25.7 mg/L), and enhanced β-carotene production in strain YL13 (211.3 mg/L, Fig. 2B).

Typically, lipophilic β-carotene is predominantly stored in the cell membrane, which drastically diminishing membrane flexibility and inducing cytotoxicity in cells [35,36]. Therefore, to provide more intracellular lipid storage in the engineered cells we overexpressed Acetyl-CoA carboxylase (*ACC1*) in the strain YL13. Surprisingly β-carotene production decreased by strain YL14 (182 mg/L, Fig. 2C), which this decrease in the production of β-carotene may attributed with directing carbon flux from carotenoids production to lipid synthesis. Conversely, the introduction of a copy of diacylglycerol acyltransferase 1 (*DGA1*) into strain YL13 resulted in a slight increase in β-carotene produced by strain YL15 (228.2 mg/L, Fig. 2C).

To enhance nicotinamide adenine dinucleotide phosphate hydrogen (NADPH) regeneration, key genes responsible for encoding glucose-6-phosphate dehydrogenase (*Zwf1*) and 6-phosphogluconate dehydrogenase (*Gnd1*) in strain YL15 were upregulated, thereby resulting in the production of β-carotene titer of 258.3 mg/L in strain YL16 (Fig. 2C). Further increase in the copy numbers of *Gnd1* to two copies increased β-carotene production in strain YL17 (284.5 mg/L, Fig. 2C). Surfactant treatment has demonstrated an enhancement in cell permeability, resulting in the release of intracellular products and enhancing overall productivity in *Y. lipolytica* [37]. For this purpose, the impact of dodecane and Triton X-100 on carotenoid production in the strain YL11 was investigated. The addition of 15 % dodecane and 4 % Triton X-100 increased β-carotene production to 213 and 186.9 mg/L, respectively (Fig. 2D and E). While introducing 5 % of olive oil increased β-carotene production to 370.6 mg/L (Fig. 2F) in the strain YL11.

### 3.2 β-carotene production is regulated by modular assembly of vital enzymes and morphological transformation

**Fig. 3:**
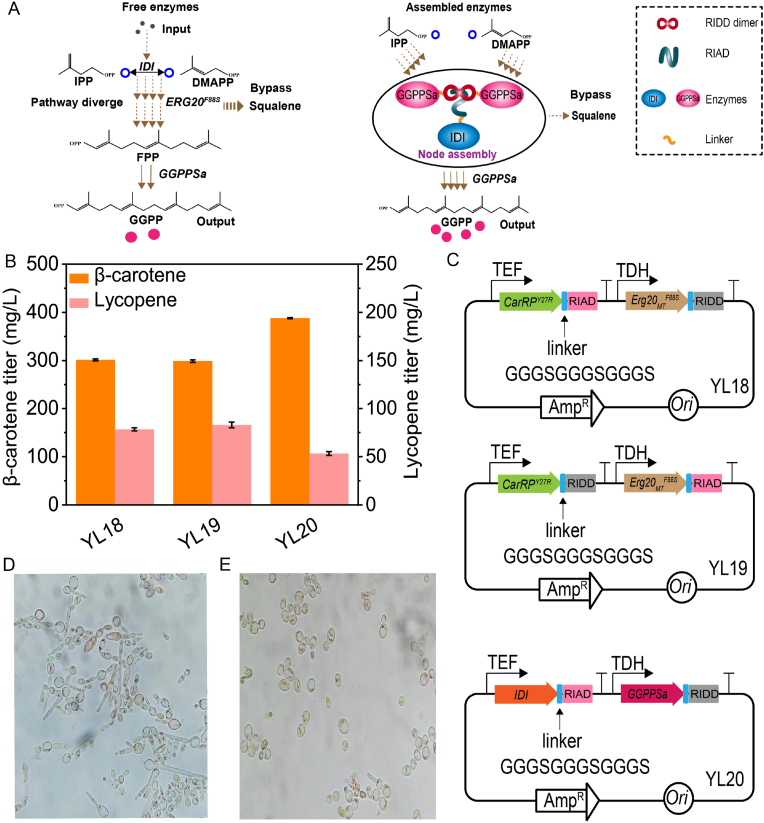
Enhancing β-carotene production by modular assembly of vital enzymes. (A) Schematic illustration of assembled enzymes, *IDI*, and GGPP synthase from *S. acidocaldarius*. (B) Effect of applying modular assembly of vital enzymes on β-carotene production. (C) Figures illustrating the vectors involved in the formation of assemblies between vital enzymes, *CarRP*^*Y27R*^-*Erg20*_*MT*_^*F88S*^, and *IDI*-GGPP synthase from *S. acidocaldarius*. (D) A microscopic image of strain YL20. (E) A microscopic image of strain YL21-Δ*mhy1*. The bar heights represent the mean of three independent experiments and the error bars represent the standard deviations.

To further enhance β-carotene production and decrease precursors diffusion, multienzyme complexes were generated using peptide tags (RIAD and RIDD). RIAD specifically binds to the dimer of RIDD, thereby resulting in the formation of a linkage that can connect any pair of enzymes to both RIAD and RIDD (Fig. 3A) [27]. Two modular enzyme assembly approaches were employed. In the first approach, *CarRP*^Y27R^ was assembled with *Erg20*_MT_^*F88S*^ in different combinations using RIDD and RIAD peptide linkers and introduced into YL17 to obtain YL18 and YL19, respectively. β-carotene production in the resulting strains did not increase substantially (Fig. 3B). In the second approach, *IDI* was assembled with *GGPPSa* leading to generation of a modular assembly known as *IDI*-RIAD::*GGPPSa*-RIDD, Introduction of this assembled enzymes into YL17 increased β-carotene production in strain YL20 to 388 mg/L (Fig. 3B).

As a distinctive dimorphic yeast, the transition of *Y*. *lipolytica* to the hyphal form is observed as a behavioral response to environmental disturbance [38,39]. Elevation in the β-carotene level induced substantial changes in the cell morphology of the engineered strain YL20 (Fig. 3D), which is potentially mediated by the gene, *MHY1* [40]. Deletion of *MHY1* in the strain YL20 resulted in an increase in β-carotene production in YL21 by 23.7 mg/L to 411.7 mg/L and almost all cells were in the form of yeast (Fig. 3E).

### 3.3 Screening of β-carotene ketolases and β-carotene hydroxylases

**Fig. 4:**
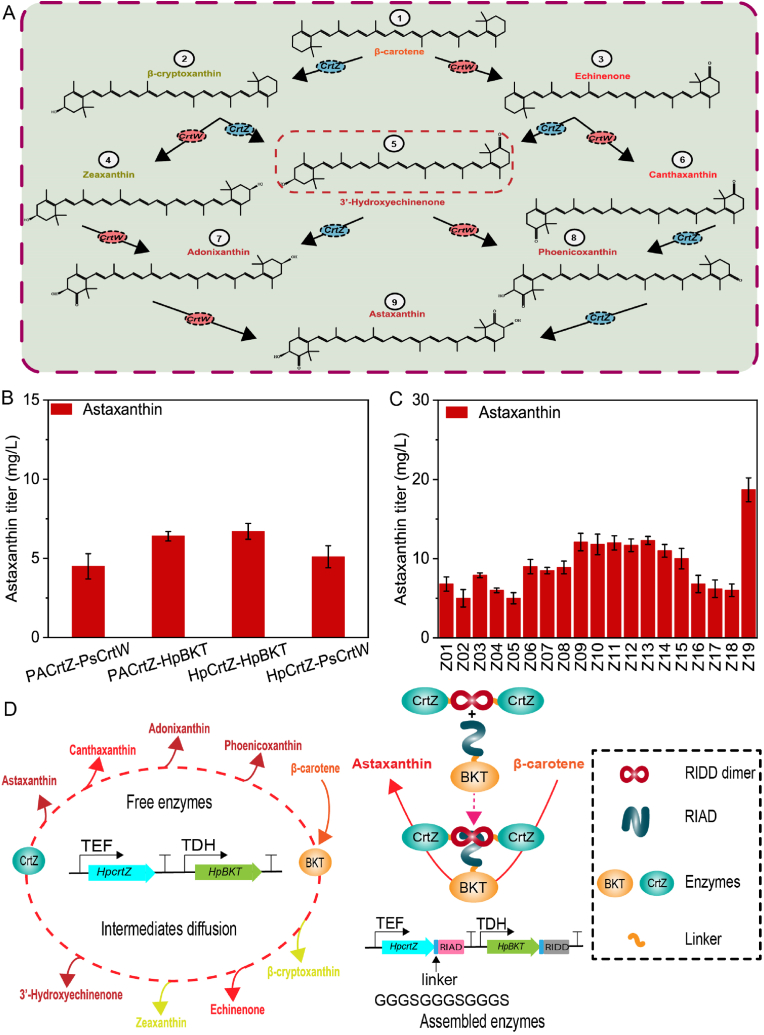
A designed astaxanthin biosynthetic pathway in the engineered *Y. lipolytica*. (A) According to the sequence in which *CrtZ* and *CrtW* regulate hydroxylation and ketolation, astaxanthin is produced from β-carotene through formation of distinct intermediates (indicated by numbers). *CrtW*; β-carotene ketolase, *CrtZ*; β-carotene hydroxylase. (B) Evaluation of the capacities of β-carotene ketolase and β-carotene hydroxylase to produce astaxanthin from various species in strain YL21. (C) Integration of *HpBKT* and *HpCrtZ* into β-carotene-producing strains and applying modular enzyme assembly to strain Z19. (D) A schematic illustration of assembled β-carotene ketolase and β-carotene hydroxylase from *H. pluvialis* involved in the astaxanthin biosynthetic pathway. The bar heights represent the mean of three independent experiments and the error bars represent the standard deviations.

Converting β-carotene to astaxanthin requires a couple of enzymes, such as β-carotene ketolase and β-carotene hydroxylase. However, the conversion process leads to the formation of eight intermediates (Fig. 4A). Strain YL21 was used to compare the effect of incorporating β-carotene ketolase (*HpBKT*) and β-carotene hydroxylase (*HpCrtZ*) coding genes from the algal, *H. pluvialis*, β-carotene ketolase (*PsCrtW*) from the bacterium, *Paracoccus* sp., and β-carotene hydroxylase (*PACrtZ*) from the bacterium *Pantoea agglomerans* in different combinations: *PACrtZ*-*PsCrtW*, *PACrtZ*-*HpBKT*, *HpCrtZ*-*HpBKT*, and *HpCrtZ*-*PsCrtW*. The resulting strains produced only 4.5, 6.4, 6.7, and 5.1 mg/L of astaxanthin, respectively (Fig. 4B).

**Fig. 5:**
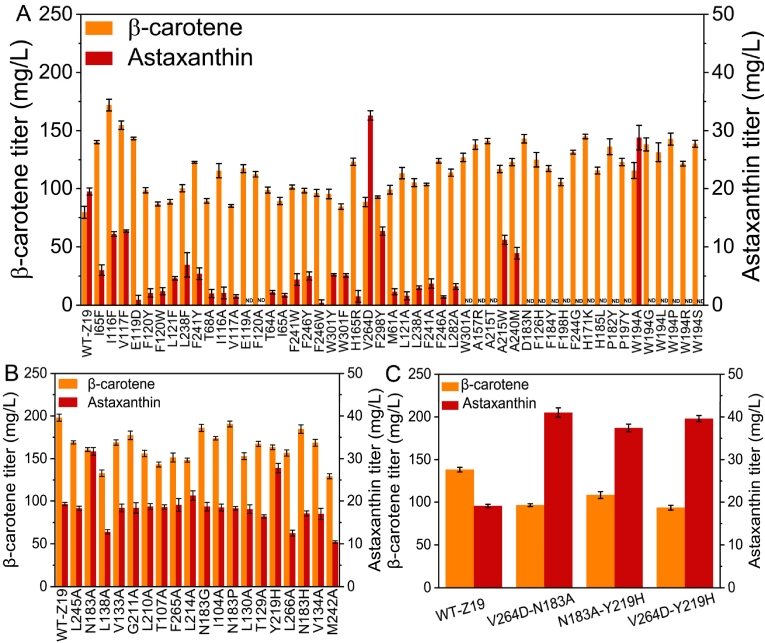
Effect of β-carotene ketolase and hydroxylase mutations on astaxanthin production. (A) Alanine scanning and changing the amino acid residues inside the pocket of β-carotene ketolase into polar residues. (B) Astaxanthin production in L214A, N183A, and Y219H increased by 2.0, 12.4, and 8.5 mg/L, respectively, under the catalytic action of β-carotene hydroxylase. (C) A combination of beneficial variants in β-carotene hydroxylase and β-carotene ketolase substantially improved astaxanthin production. The bar heights represent the mean of three independent experiments and the error bars represent the standard deviations.

Production of small portion of astaxanthin and accumulation of large amounts of the precursor, β-carotene by the engineered strains prompted the integration of astaxanthin-related genes from *H. pluvialis* into the β-carotene-producing strains YL03–YL21 to construct strains Z01–Z18, respectively (Fig. 4C), to evaluate whether accumulation of β-carotene shows any adverse effect on conversion rate from β-carotene to astaxanthin. Among the engineered strains, Z13 produced the highest amount of astaxanthin (12.3 mg/L). However, the integration of *PACrtZ*-*PsCrtW*, *PACrtZ*-*HpBKT*, and *HpCrtZ*-*PsCrtW* into YL15 resulted in the production of 8.4, 11.6, and 8.6 mg/L of astaxanthin, respectively. The reconstruction of *HpBKT* and *HpCrtZ* as *HpCrtZ*-RIAD::*HpBKT*-RIDD (Fig. 5B) using a modular enzyme assembly approach led to the production of 18.7 mg/L of astaxanthin by strain Z19 (Fig. 4C). This result shows the effectiveness of modular enzyme assembly in decreasing the distribution of various intermediates in the metabolic pathways and enhancing the accumulation of the target products. To enhance production and reduce the impact of astaxanthin accumulation in the engineered strain, the impact of dodecane (Fig. S1A), Triton X-100, and olive oil (Fig. S1B) on astaxanthin production in the strain Z19 was investigated. Addition of 1 % Triton X-100 into the culture media increased astaxanthin production to 28.8 mg/L (Fig. S1C).

### 3.4 Enhancing astaxanthin production through engineered enzymes

**Fig. 6:**
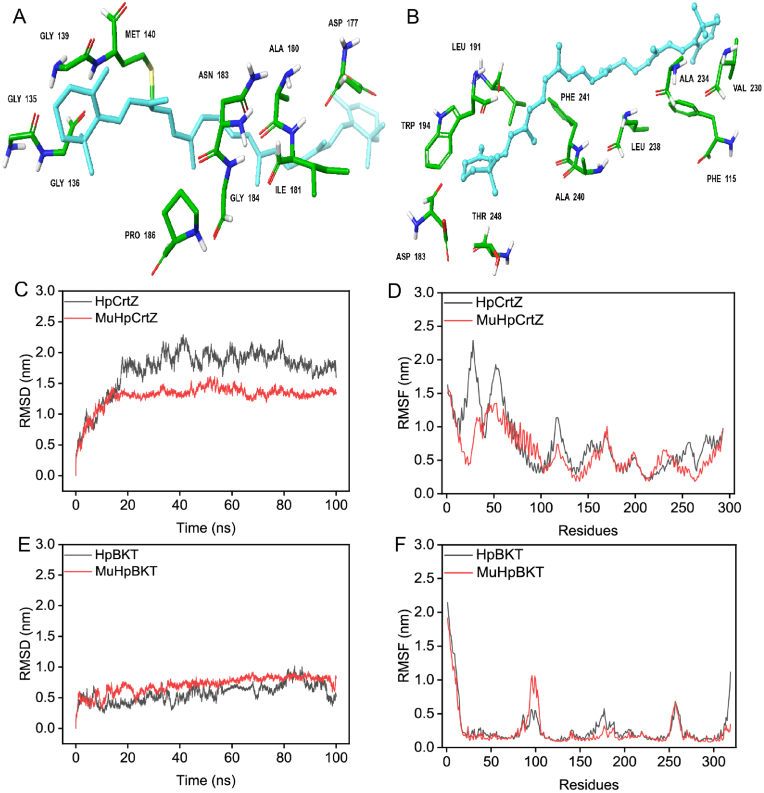
Docking and 100-ns MD simulations for *HpCrtZ* and *HpBKT*. (A) Enzyme-substrate docking of *HpCrtZ*. (B) Enzyme-substrate docking of *HpBKT*. RMSD (C) and RMSF (D) analysis of *HpCrtZ*. RMSD (E) and RMSF (F) analysis of *HpBKT*. The ligand β-carotene is shown in a light blue rod shape. RMSD and RMSF results for wild-type *HpCrtZ* and *HpBKT* are represented in black. RMSD and RMSF results for mutant *HpCrtZ* and *HpBKT* are represented in red. RMSD, root mean square deviation; RMSF, root mean square fluctuations.

The accumulation of a high quantity of β-carotene in the astaxanthin producing strain Z19 indicated that the conversion of β-carotene to astaxanthin posed a challenge to the pathway. Therefore, the catalytic activity of the fused enzymes increased through a directed evolution approach. Structural assessment and computational docking approaches were used to analyze 91 mutants and the wild-type β-carotene ketolase (*BKT*) (Fig. 6B). A particular variant, W194A was determined, which led to a marked increase in astaxanthin production by 10.1 mg/L, in turn, yielding 28.7 mg/L. The introduction of the triple mutation V264D/F298Y/H165R by Zhou et al. resulted in a 2.4-fold increase in *HpBKT* activity in *S*. *cerevisiae* [41], and only V264D exhibited an increase in astaxanthin production in *Y. lipolytica* by 13.9 mg/L, in turn, yielding 32.6 mg/L (Fig. 5A). Regarding β-carotene hydroxylase (*CrtZ*) (Figs. 6A), 19 variants were generated. Among the mutants, L214A boosted astaxanthin production by only 2 mg/L (Fig. 5B), while N183A and Y219H showed a considerable enhancement in astaxanthin synthesis by 12.4 and 8.5 mg/L, respectively, in turn, reaching 31.7 and 27.8 mg/L, respectively (Fig. 5B). Combination between best variants of β-carotene hydroxylase and β-carotene ketolase in V264D position further enhanced astaxanthin production to 41.0 mg/L (Fig. 5C).

Subsequently, 100-ns MD simulations were performed for *HpCrtZ* and *HpBKT* using substrate complexes. For *HpCrtZ* and *HpCrtZ* mutations, the root mean square deviation (RMSD) results (Fig. 6C) showed that the binding state between the mutants and the substrate tended to stabilize after 20 ns, while the binding state between the wild type and the substrate was more unstable. The root mean square fluctuation (RMSF) results (Fig. 6D) showed that the rigidity of the *HpCrtZ* mutants was higher than that of the wild type. Similarly, the RMSD (Fig. 6E) and RMSF (Fig. 6F) values for *HpBKT* and *HpBKT* mutations showed that the mutated catalytic pocket binds more tightly to the substrate, thereby resulting in higher activity.

### 3.5 Multicopy integration with subcellular expression of engineered and assembled enzymes enhances astaxanthin production

**Fig. 7:**
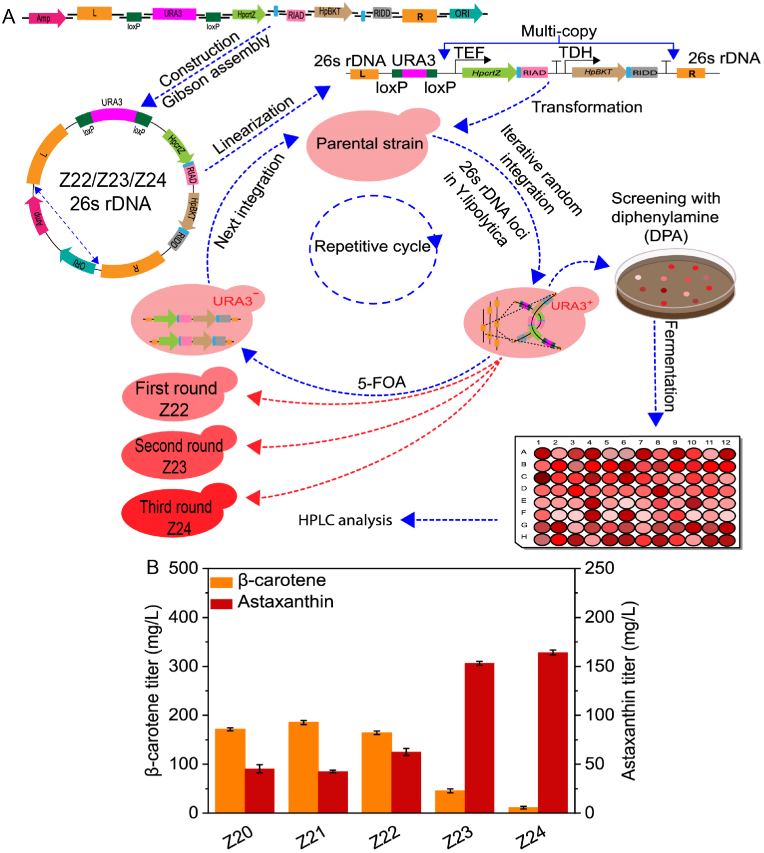
A schematic illustration of the engineered *Y. lipolytica* for random multicopy integration. (A) The 26s rDNA randomly integrated genes into the *Y. lipolytica* genome at unspecified locations. The positive transformants were grown on the uracil dropout yeast nitrogen base (YNB) plate. Diphenylamine (DPA) was used for screening. Colonies that produced astaxanthin were tested in yeast extract peptone dextrose (YPD) medium through fermentation, with the colony producing the highest amount of astaxanthin being employed in next rounds of multicopy integration. (B) Multicopy integration of the engineered and assembled *HpCrtZ* and *HpBKT* increased astaxanthin production. The bar heights represent the mean of three independent experiments and the error bars represent the standard deviations.

Even though astaxanthin was effectively synthesized in the modified *Y. lipolytica*, to develop strains with enhanced β-carotene flux to astaxanthin, we investigated the impact of increasing the copy numbers of astaxanthin-related genes for multiple round integration into rDNA loci (Fig. 7A). First, we introduced *HpCrtZ* and *HpBKT* separately to engineer strains Z20 and Z21, respectively. The resulting strain Z20 exhibited an increase in astaxanthin synthesis (45.3 mg/L), while strain Z21 did not exhibit a notable increase in astaxanthin production (Fig. 7B).

Then, the engineered and assembled enzymes were incorporated into rDNA loci for multiple rounds. The first round of integration (Z22) yielded 62.5 mg/L of astaxanthin and accumulated a substantial amount of the precursor substance, β-carotene (164.2 mg/L, Fig. 7B). Afterward, we increased the rounds of multicopy integration of the engineered enzymes. In the second round (Z23), the astaxanthin titer increased to 153.1 mg/L (Fig. 7B), while the third round (Z24) further boosted astaxanthin production to 164.3 mg/L (Fig. 7B).

**Fig. 8:**
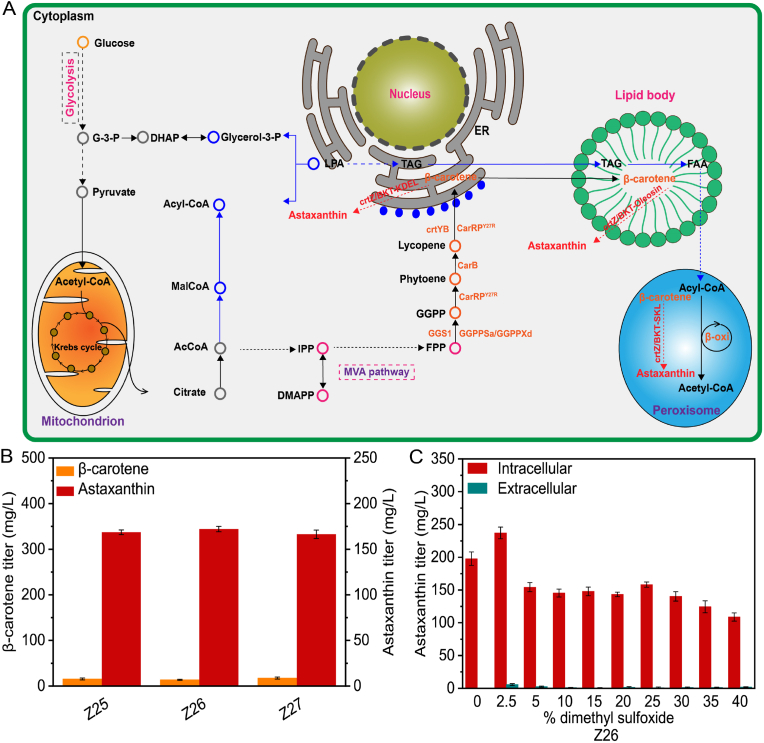
Enhanced astaxanthin biosynthesis with subcellular *HpCrtZ* and *HpBKT* expression. (A) AcCoA, Acetyl-CoA; MalCoA, Malonyl-CoA, DHAP, dihydroxyacetone phosphate; TAG: triacyl-glycerol; *CarRP*^*Y27R*^, bifunctional phytoene synthase/lycopene β-cyclase from *M. circinelloides* after specific point mutation; *CarB*, phytoene dehydrogenase from *M*. *circinelloides*; ER, endoplasmic reticulum. Genes highlighted in red denote enzymes involved in astaxanthin production by heterologous subcellular engineering. More than one catalytic stage is shown in dotted arrows. (B) Subcellular expression of *HpCrtZ* and *HpBKT* enhanced astaxanthin biosynthesis. (C) Astaxanthin titers following treatment with various doses of dimethyl sulfoxide. The bar heights represent the mean of three independent experiments and the error bars represent the standard deviations.

Astaxanthin production was further enhanced by directing the engineered and assembled enzymes to various subcellular compartments, including the ER, lipid bodies, and peroxisomes (Fig. 8A) in the strain Z24 to obtain strains Z25, Z26, and Z27, respectively, using specific signal peptides described in a previous study [34]. Strain Z26, which targeted to lipid bodies, yielded an astaxanthin titer of 172.1 mg/L (Fig. 8B), indicating an increase of 7.8 mg/L. Strains Z25 and Z27, which targeted to ER and peroxisomes, yielded astaxanthin titers of 168.5 and 166.3 mg/L, respectively (Fig. 8B).

### 3.6 Fed-batch fermentation for astaxanthin production in 5-L bioreactor

Auxotrophic markers, *LEU2* and *URA3* are essential elements for the growth of the modified strains; therefore, we restored both auxotrophic markers in the final strain (Z26), which improved astaxanthin production to 198.0 mg/L. It has been reported DMSO promotes the fluidity of the cell membrane which resulting in notable improvement in the membrane permeability [42]. Therefore, to reduce the effects of astaxanthin accumulation on the cells, we evaluated the impact of various DMSO concentrations on astaxanthin production. The results revealed that 2.5 % DMSO substantially increased astaxanthin production by 39.1 mg/L and the titer reached 237.1 mg/L (Fig. 8C), which was 1.7-fold higher than the titer reported previously [43].

**Fig. 9:**
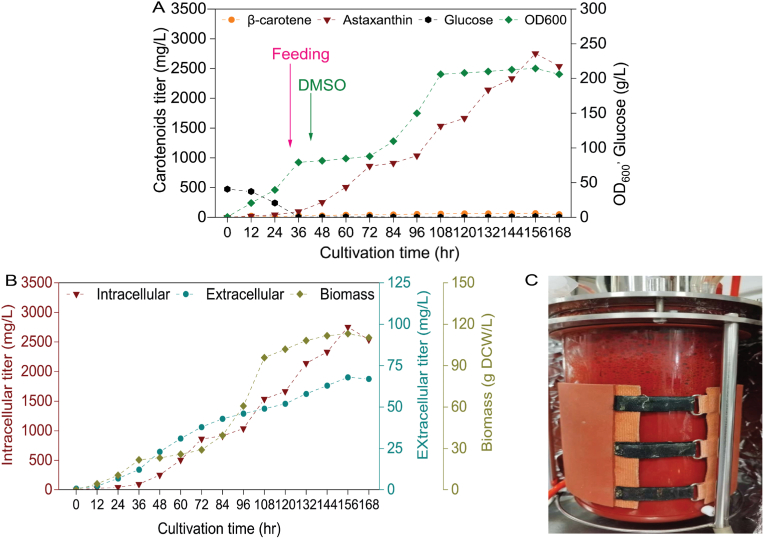
Astaxanthin production in a 5-L bioreactor by strain Z26. (A) Astaxanthin was produced in a 5-L bioreactor with the addition (2.5 %) of dimethyl sulfoxide (DMSO). (B) Biomass, titer of extracellular and intracellular astaxanthin produced during fermentation process. Pink arrow indicates the time point at which feeding began. Green arrow indicates the time point at which DMSO was introduced into the bioreactor. (C) 5-L Bioreactor during fed-batch fermentation of strain Z26.

Finally, to evaluate the potential of the modified strains in the synthesis of astaxanthin, fed-batch fermentation was conducted for the strain Z26 with the addition of 2.5 % DMSO after 40 h of fermentation. Intracellular astaxanthin production reached 2752 mg/L (113 g DCW/L) and 68 mg/L of extracellular astaxanthin was produced at 156 h of fermentation (Fig. 9A and B). Crucially, this advanced synthesis of astaxanthin was accomplished with solely cost-effective mineral fermentation media. In the natural producers *H. pluvialis* and *X. dendrorhous,* astaxanthin productivity is estimated to range from 21 to 46 mg/L/d, respectively [44,45], while the Z26 strain achieved an average productivity of 423.4 mg/L/d during the fermentation process, which was 20 and 9 times higher than productivity in *H. pluvialis* and *X. dendrorhous*, respectively. Additionally, achieved titer in this study was 1.5 times higher than the highest reported titer in *E. coli* [3]. Regarding the engineered yeast platforms for astaxanthin production, the achieved productivity by strain Z26 was 1.5 times higher than previously reported productivity in *Y. lipolytica* [20], about six times than productivity in *S. cerevisiae* [18], 31 and 1270 times higher than recently achieved productivity in *Pichia pastoris* [46] and *Kluyveromyces marxianus*, respectively [2].

## 4 Discussion

Biotechnological production of astaxanthin is a favorable substitute for extracting this pigment from its natural sources or synthesizing it chemically. However, the efficacy of microbial production of astaxanthin is limited by a low availability of the precursor molecules and low catalytic activity of the essential enzymes involved in the biosynthetic pathways [47]. In the present study, we engineered *Y*. *lipolytica* to optimize astaxanthin synthesis by implementing several strategies, such as boosting the biosynthesis of the precursor substance β-carotene, increasing the catalytic activity of key enzymes (i.e., β-carotene ketolase and β-carotene hydroxylase) through a directed evolution approach, decreasing intermediate diffusion in the synthetic pathways by applying modular enzyme assembly along with increasing copy numbers of the genes through integration at rDNA loci, and directing the astaxanthin biosynthetic pathway into sub-organelles.

To substantially buildup target products, adequate quantities of precursor substances are necessary [48]. β-Carotene serves as a vital precursor for astaxanthin synthesis [49]. In a previous study, 7.6 g/L of β-carotene was produced through regulation of morphological transition in *Y. lipolytica* [39]. Ma et al. recently produced 39.5 g/L of β-carotene through elimination of lycopene substrate inhibition [50]. In this study, a *Y. lipolytica* strain was engineered for β-carotene synthesis by introducing heterologous β-carotene biosynthetic genes from *M. circinelloides*, enhancing the MVA pathway, truncating the promotor of *ERG9* to 50 base pairs, increasing NADPH regeneration, as well as controlling morphological transition that accumulated 411.7 mg/L of β-carotene. Transforming β-carotene to astaxanthin is considerably influenced by the origins of ketolase and hydroxylase enzymes, and the catalytic activity of enzymes that produce astaxanthin from different species has been evaluated in numerous studies [16,[51], [52], [53], [54]]. Among the enzymes investigated in this study, β-carotene ketolase and β-carotene hydroxylase from *H. pluvialis* produced the highest amount of astaxanthin (12.3 mg/L). The fusion of *CrtZ* and *CrtW* has been shown to enhance astaxanthin production in *Y. lipolytica* and *Corynebacterium glutamicum* [20,55]. Construction of *HpCrtZ*-*HpBKT* as a single enzyme complex effectively restricted intermediate diffusion and enhanced the final product yield to 18.7 mg/L.

Currently, limited catalytic activities of ketolase and hydroxylase enzymes are major obstacles in astaxanthin biosynthesis by microbial chassis. Astaxanthin production in *E. coli* was enhanced by 5.35-fold and 69 % through *CrtW* engineering [56,57]. Ding et al. increased astaxanthin content in *S. cerevisiae* by 3.8-fold through a directed evolution approach of the fused *CrtZ* and *CrtW* [58]. Zhou et al. improved the activity of ketolase enzyme in *S. cerevisiae* by 2.4-fold [41]. Here, 19 mutants of *HpCrtZ* and 91 mutants of *HpBKT* have been generated by subjecting hydroxylase and ketolase enzymes to a directed evolution approach. N183A and Y219H variants in β-carotene hydroxylase increased astaxanthin production by 2.5 and 2.2 times higher than the parental strain, while 1.6 and 1.4 times higher than the fused enzymes. In β-carotene ketolase, W194A and V264D variants produced astaxanthin 2.3 and 2.6 times higher than the original strain, respectively. A combination of beneficial variants V264D + N183A yielded a higher astaxanthin titer (41 mg/L) than individual mutants V264D (32.6 mg/L) and N183A (31.7 mg/L), which shows the effectiveness of combining beneficial mutants for astaxanthin production.

Despite the progress made in astaxanthin production, β-carotene is still accumulated in the engineered strain. Zhu et al. recently increased the conversion rate of β-carotene to astaxanthin through random integration of 20 copies of *HpBKT* and *HpCrtZ* at rDNA loci [20]. To overcome this challenge, astaxanthin synthesis was further improved to 164.3 mg/L through multicopy integration at rDNA loci, which was 13.3-fold higher than the parental strain (Figs. S4A, B, and C). β-carotene synthesis is speculated to occur in the ER [59]. Sequestration of β-carotene inside lipid droplets forms a spatial barrier, which makes it inaccessible for conversion to astaxanthin by cytosolic ketolase and hydroxylase enzymes [1,43,60]. In addition to lipid bodies, peroxisomes serve as another compartment that can affect accumulation of β-carotene within cells [61]. Therefore, we localized the astaxanthin biosynthetic pathway to the lipid bodies, peroxisome, and ER using specific localization signals [34,43,49]. Localization of the engineered enzymes increased astaxanthin production to 172.1 mg/L in the lipid droplet targeted strain. The final constructed strain (Z26) produced the highest reported productivity (434 mg/L/d) up to date in *Y. lipolytica* after restoration of the auxotrophic markers and performing fed-batch fermentation using inorganic salt medium.

## 5 Conclusion

In this study, *Y. lipolytica* was genetically modified for the biosynthesis of β-carotene and subsequently astaxanthin. We discovered modular enzyme assembly as a pivotal factor in the production of β-carotene and decreasing intermediates diffusion in the astaxanthin synthetic pathway. We also increased the catalytic activity of assembled ketolase and hydroxylase enzymes through a directed evolution approach and using a cheap inorganic salt medium for high-level synthesis of 3*S*,3′*S* astaxanthin through fed-batch fermentation. For future investigations, identifying more effective enzymes involved in astaxanthin production, suppressing competitive pathways, introducing exogenous pathways, such as isoprenol utilizing pathway for isopentenyl pyrophosphate enhancement could enhance the performance of strains associated with astaxanthin production in *Y. lipolytica*.

## CRediT authorship contribution statement

**Chalak Najat Abdullah:** Writing – review & editing, Writing – original draft, Visualization, Investigation. **Mengsu Liu:** Investigation. **Qihang Chen:** Investigation. **Song Gao:** Writing – review & editing, Funding acquisition. **Changtai Zhang:** Writing – review & editing. **Shike Liu:** Writing – review & editing. **Jingwen Zhou:** Writing – review & editing, Supervision, Funding acquisition.

## Declaration of competing interest

The authors declare that they have no known competing financial interests or personal relationships that could have appeared to influence the work reported in this paper.

## Appendix A Supplementary data

Multimedia component 1 Plasmids used in this study (Table S1), Codon optimized nucleotide sequences of the heterologous genes used in this study (Table S2), primers used in this study (Table S3), strains used in this study (Table S4). Impact of different supplements on astaxanthin production (Fig. S1). Effect of butylated hydroxytoluene (BHT) on carotenoids production (Fig. S2). Effect of site saturation mutation of β-carotene ketolase on astaxanthin production (Fig. S3). Enhancement of astaxanthin production through multicopy integration at 26s rDNA loci (Fig. S4). HPLC chromatograms of carotenoid standards and carotenoids produced by strain Z22 (Fig. S5). LC-Q-TOF/MS spectra of astaxanthin standard and Z22 sample (Fig. S6). Fluorescence detection of mCherry with different localization signals (Fig. S7). Schematic representation of genetic constructs used to construct strain Z26 (Fig. S8). Chiral HPLC analysis of the astaxanthin generated by strain Z26 (Fig. S9). Illustration of strain Z26 production stability according to the number of generations obtained in YPD medium (Fig. S10). Multimedia component 1
