# Supplementary material for: Efficient production of astaxanthin in Yarrowia lipolytica through metabolic and enzyme engineering
Source: Synth Syst Biotechnol. 2025 Feb 28;10(3):737–50. doi: 10.1016/j.synbio.2025.02.014 (PMC12002715; doi:10.1016/j.synbio.2025.02.014)
Supplement: Multimedia component 1 — Plasmids used in this study (Table S1), Codon optimized nucleotide sequences of the heterologous genes used in this study (Table S2), primers used in this study (Table S3), strains used in this study (Table S4). Impact of different supplements on astaxanthin production (Fig. S1). Effect of butylated hydroxytoluene (BHT) on carotenoids production (Fig. S2). Effect of site saturation mutation of β-carotene ketolase on astaxanthin production (Fig. S3). Enhancement of astaxanthin production through multicopy integration at 26s rDNA loci (Fig. S4). HPLC chromatograms of carotenoid standards and carotenoids produced by strain Z22 (Fig. S5). LC-Q-TOF/MS spectra of astaxanthin standard and Z22 sample (Fig. S6). Fluorescence detection of mCherry with different localization signals (Fig. S7). Schematic representation of genetic constructs used to construct strain Z26 (Fig. S8). Chiral HPLC analysis of the astaxanthin generated by strain Z26 (Fig. S9). Illustration of strain Z26 production stability according to the number of generations obtained in YPD medium (Fig. S10). [file mmc1.docx]

**Supplementary materials**

**Efficient production of astaxanthin in *Yarrowia lipolytica* through metabolic and enzyme engineering**

Chalak Najat Abdullah^1,2,3^, Mengsu Liu^1,3^, Qihang Chen^1,3^, Changtai Zhang^1,3^, Shike liu^1,3^, Song Gao^1,3^, Jingwen Zhou^1,3,4,5,*^

^1^ School of Biotechnology, Jiangnan University, 1800 Lihu Road, Wuxi, Jiangsu 214122, China.

^2^ Department of Biology, College of Science, University of Sulaimani, 46001 Sulaimaniyah, Kurdistan region, Iraq.

^3^ Engineering Research Center of Ministry of Education on Food Synthetic Biotechnology, Jiangnan University, 1800 Lihu Road, Wuxi, Jiangsu 214122, China.

^4^ Science Center for Future Foods, Jiangnan University, 1800 Lihu Road, Wuxi, Jiangsu 214122, China.

^5^ Jiangsu Provisional Research Center for Bioactive Product Processing Technology, Jiangnan University, 1800 Lihu Road, Wuxi, Jiangsu 214122, China.

* Corresponding author

Jingwen Zhou

Mailing address: Science Center for Future Foods, Jiangnan University, 1800 Lihu Road, Wuxi, Jiangsu 214122, China.

Phone: +86-510-85914371, Fax: +86-510-85914371

E-mail: [zhoujw1982@jiangnan.edu.cn](mailto:zhoujw1982@jiangnan.edu.cn).

**Contents**

| **Items** | **Page No.** |
| --- | --- |
| **Figure S1.** Impact of different supplements on astaxanthin production. | **4** |
| **Figure S2.** Effect of butylated hydroxytoluene (BHT) on carotenoids production. | **5** |
| **Figure S3.** Effect of site saturation mutation of β-carotene ketolase on astaxanthin production. | **6** |
| **Figure S4.** Enhancement of astaxanthin production through multicopy integration at 26s rDNA loci. | **7** |
| **Figure S5.** HPLC chromatograms of carotenoid standards and carotenoids produced by Z22 strain. | **8** |
| **Figure S6.** LC-Q-TOF/MS spectra of astaxanthin standard (A) and Z22 sample (B). | **9** |
| **Figure S7.** Fluorescence detection of mCherry without any signal (A), mCherry with KDEL (B), mCherry with SKL (C), mCherry with oleosin sequence (D). | **10** |
| **Figure S8.** Schematic representation of genetic constructs used to construct Z26 strain. | **11** |
| **Figure S9.** Chiral HPLC analysis of the astaxanthin generated by Z26 strain. | **12** |
| **Figure S10.** Illustration of Z26 strain production stability according to the number of generations obtained in YPD medium. | **13** |
| **Table S1.** Plasmids used in this study. | **14 - 26** |
| **Table S2.** Codon optimized nucleotide sequences of the heterologous genes used in this study. | **27 - 47** |
| **Table S3.** Primers used in this study. | **48 - 76** |
| **Table S4.** Strains used in this study. | **77 - 80** |
| **References** | **81** |

**
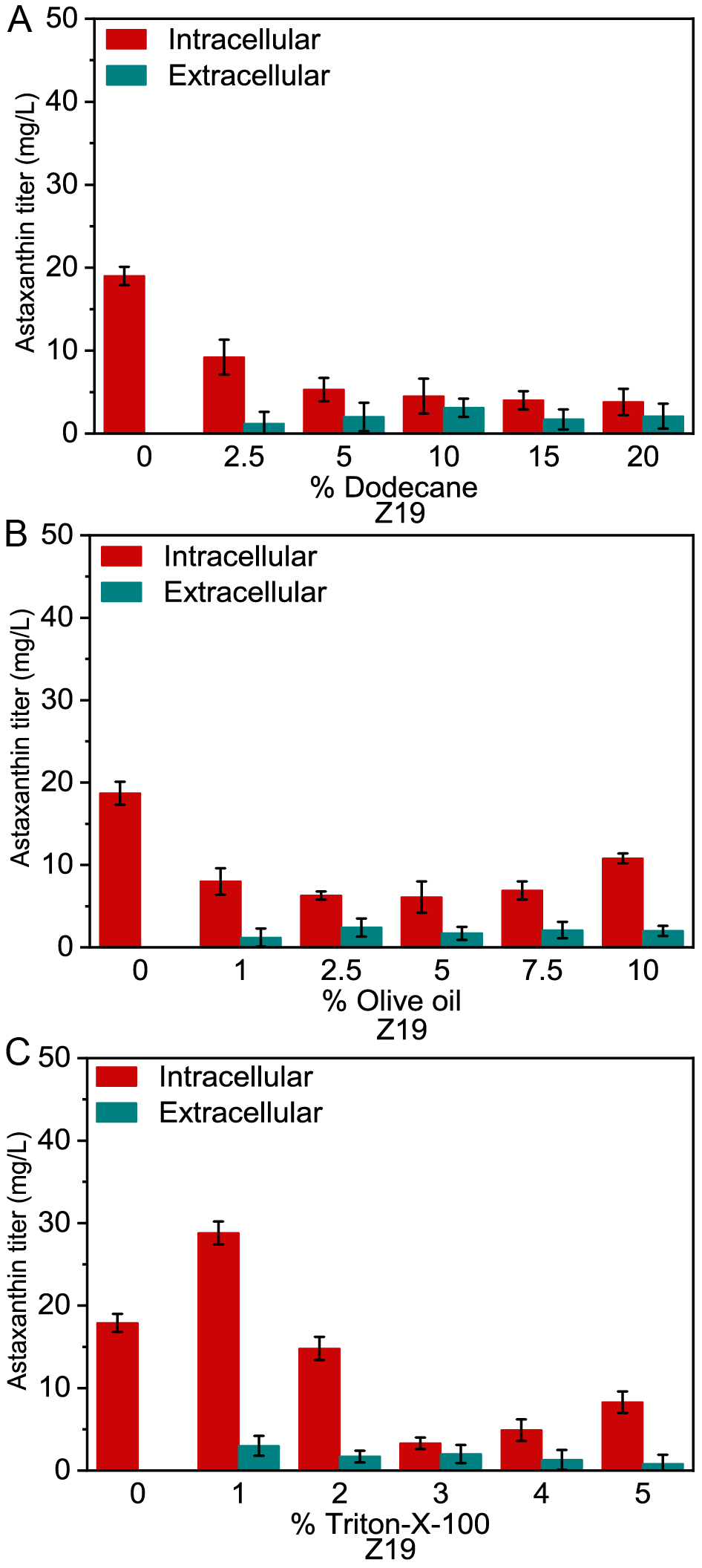
**

**Fig. S1. Impact of different supplements on astaxanthin production**.

(A) Astaxanthin titers in strain Z19 following treatment with various doses of dodecane. (B) Astaxanthin titers in strain Z19 following treatment with various doses of olive oil. (C) Astaxanthin titers in strain Z19 following treatment with various doses of Triton-X-100. The bar heights represent the mean of three independent experiments and the error bars represent the standard deviations.

**
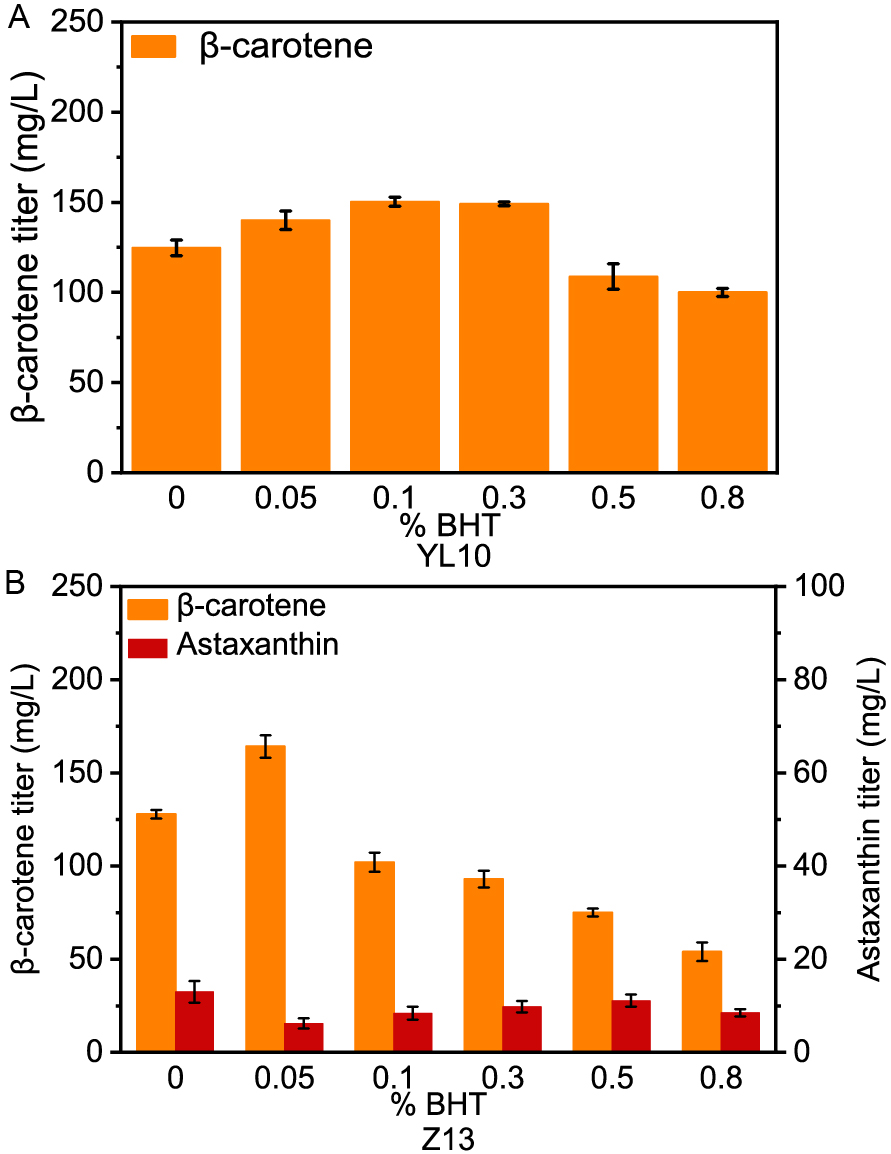
**

**Fig. S2. Effect of butylated hydroxytoluene (BHT) on carotenoids production**.

(A) β-carotene titer in strain YL10 following treatment with various doses of BHT. (B) β-carotene and astaxanthin titers in strain Z13 following treatment with various doses of BHT. The bar heights represent the mean of three independent experiments and the error bars represent the standard deviations.

**
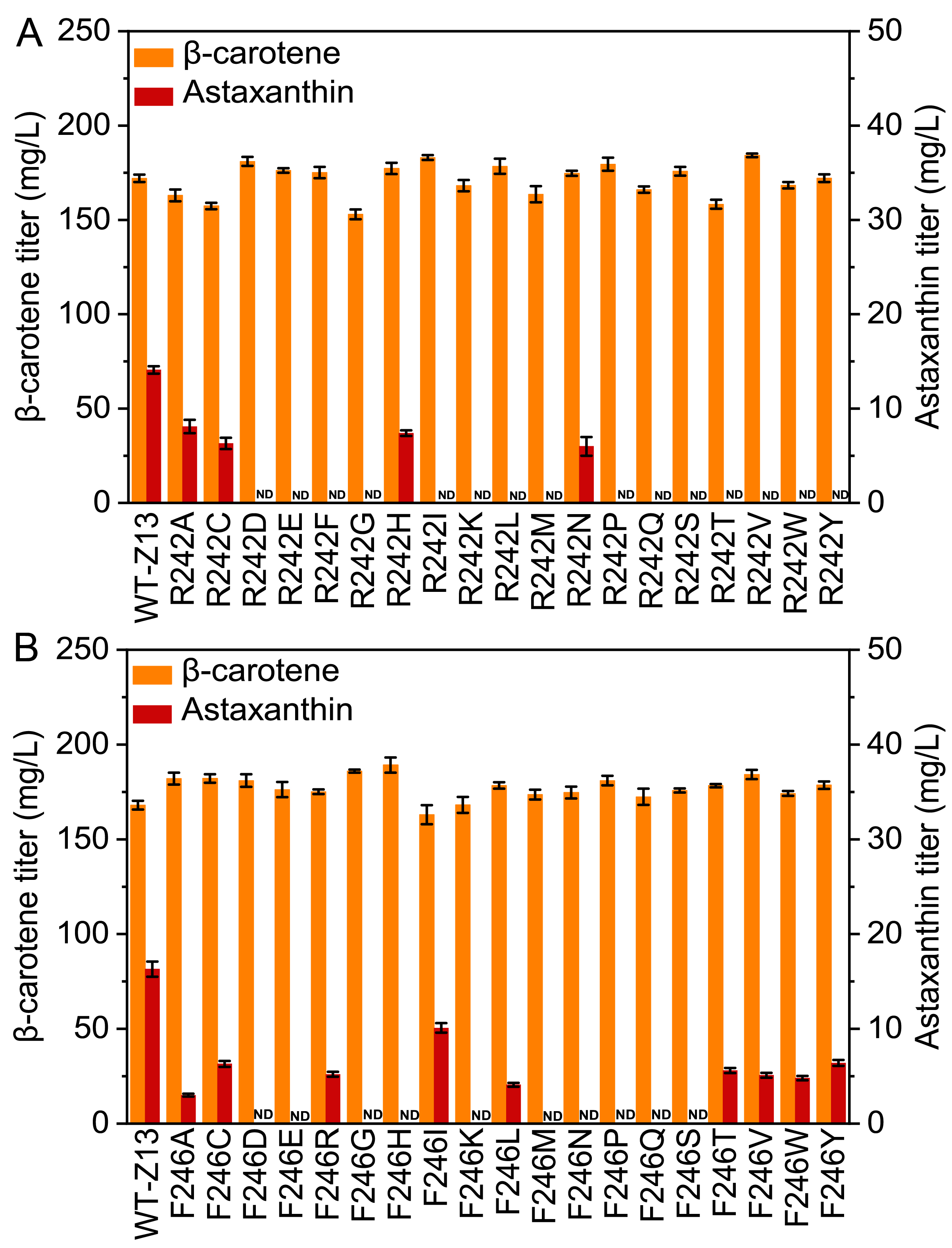
**

**Fig. S3. Effect of site saturation mutation of β-carotene ketolase on astaxanthin production.**

(A) Site-saturation mutations of β-carotene ketolase at the R242 site. (B) Site-saturation mutations of β-carotene ketolase at the F246 site. The bar heights represent the mean of three independent experiments and the error bars represent the standard deviations.

**
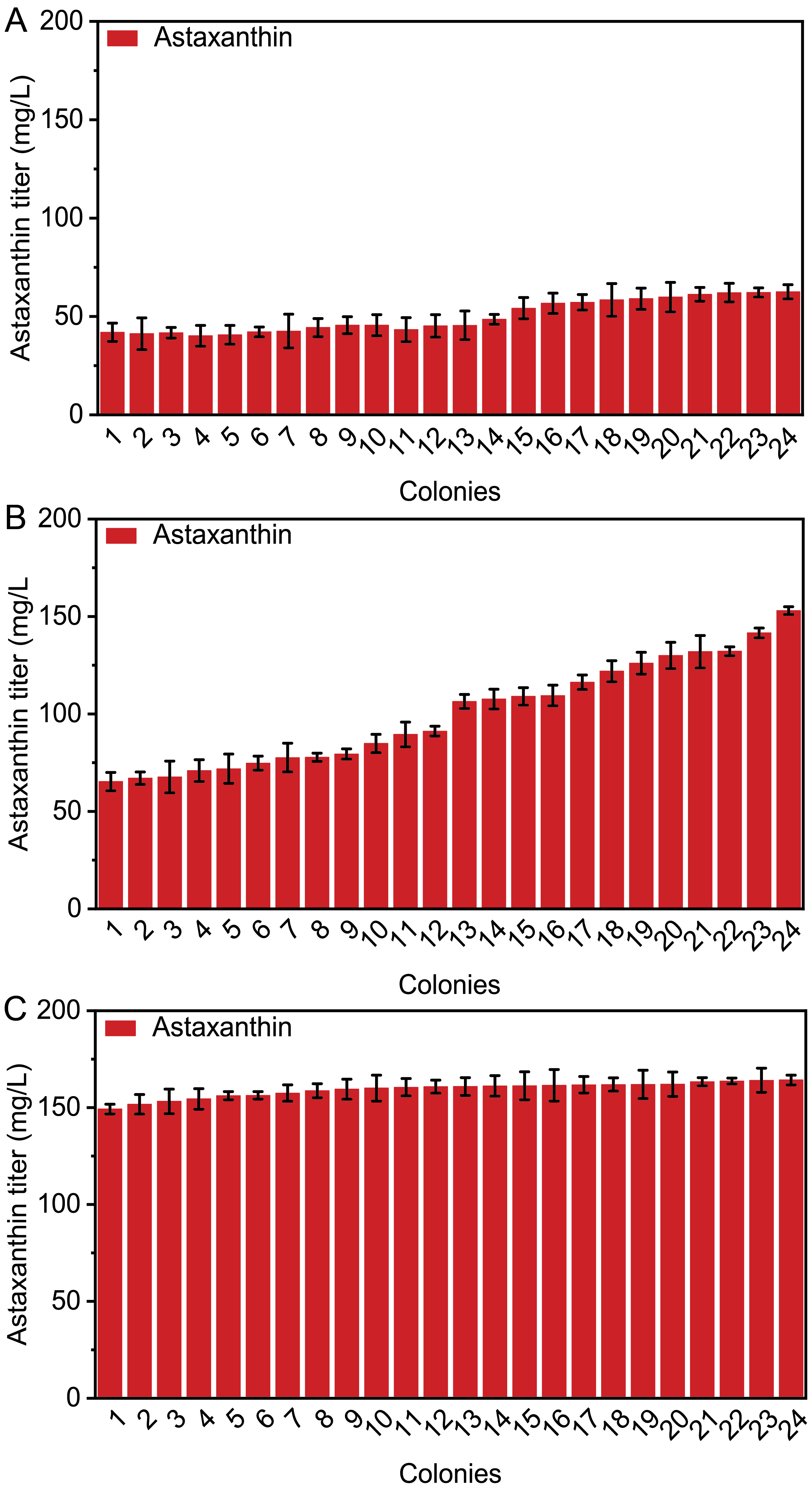
**

**Fig. S4. Enhancement of astaxanthin production through multicopy integration at 26s rDNA loci.**

(A) Selected colonies for the first round of iterative integration at 26S rDNA loci to boost astaxanthin production in the engineered *Y. lipolytica* strains. (B) Selected colonies for the second round of iterative integration at 26S rDNA loci to boost astaxanthin production in the engineered *Y. lipolytica* strains. (C) Selected colonies for the third round of iterative integration at 26S rDNA loci to boost astaxanthin production in the engineered *Y. lipolytica* strains. The bar heights represent the mean of three independent experiments and the error bars represent the standard deviations.

**
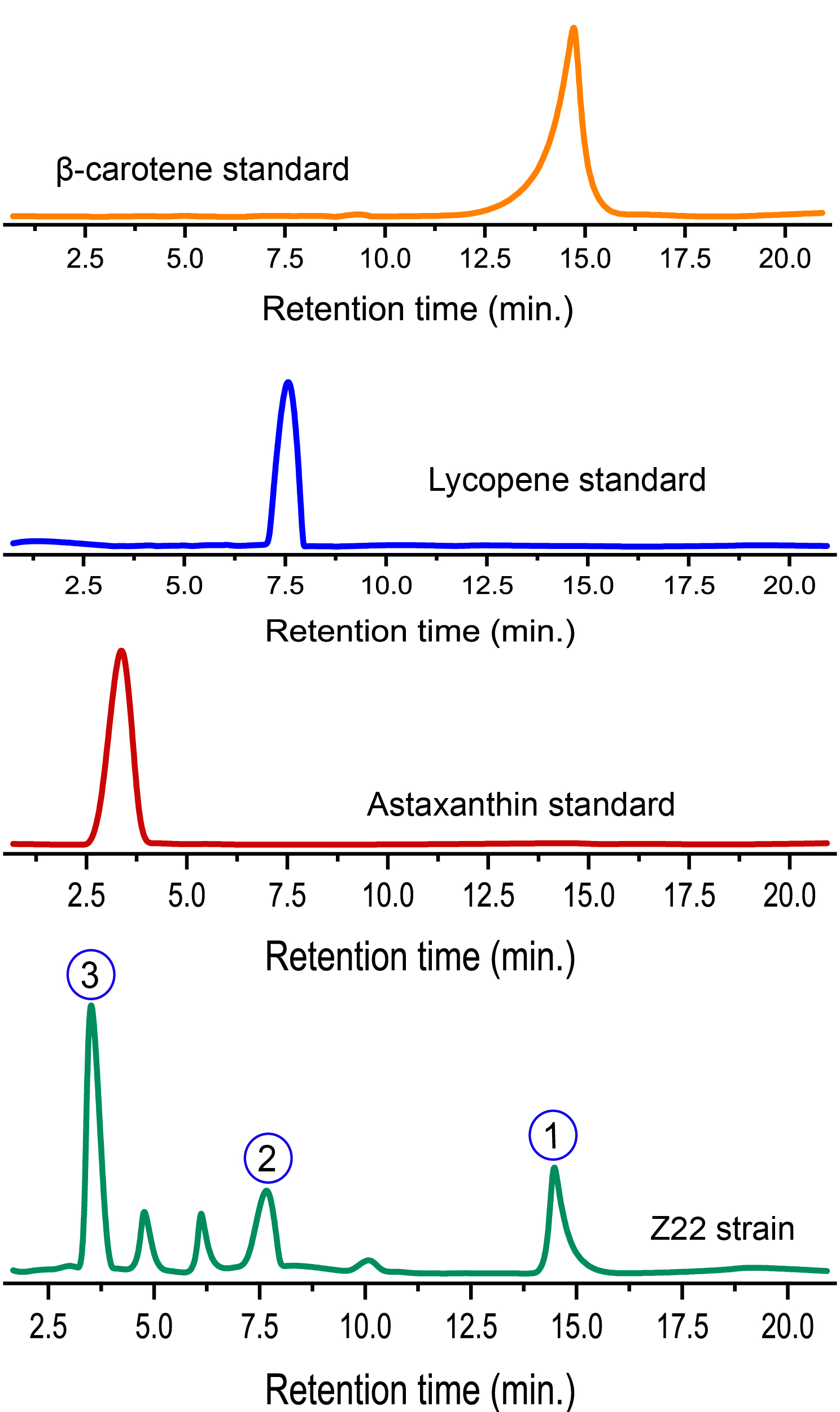
**

**Fig. S5. HPLC chromatograms of carotenoid standards and carotenoids produced by strain Z22.**

Peaks of carotenoids produced by strain Z22 are indicated by numbers in the HPLC chromatogram: 1, β-carotene; 2, lycopene; 3, astaxanthin.

**
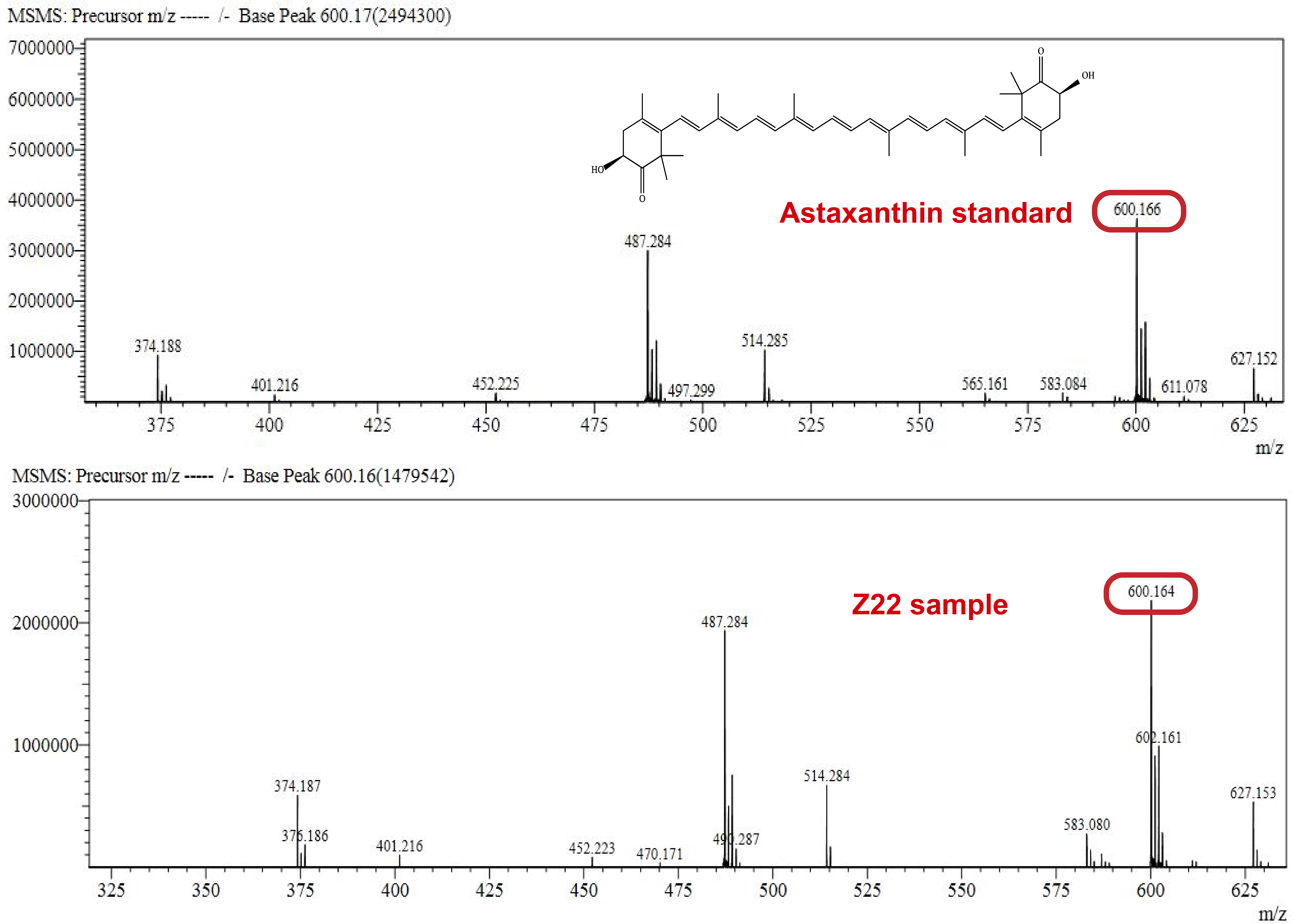
Fig. S6. LC-Q-TOF/MS spectra of astaxanthin standard (A) and Z22 sample (B).**

**
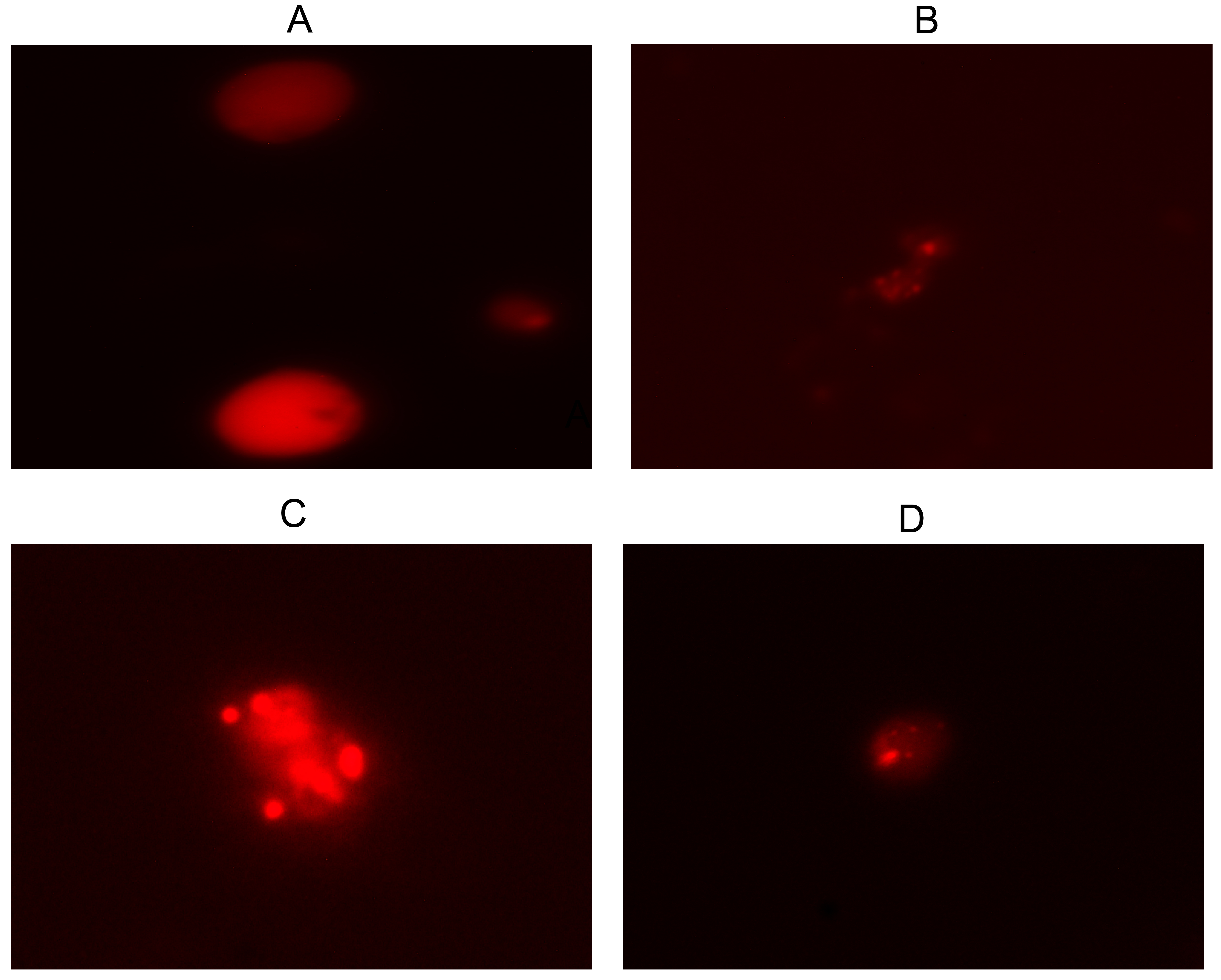
**

**Fig. S7. Fluorescence detection of mCherry without any signal (A), mCherry with KDEL (B), mCherry with SKL (C), mCherry with oleosin sequence (D).**

**
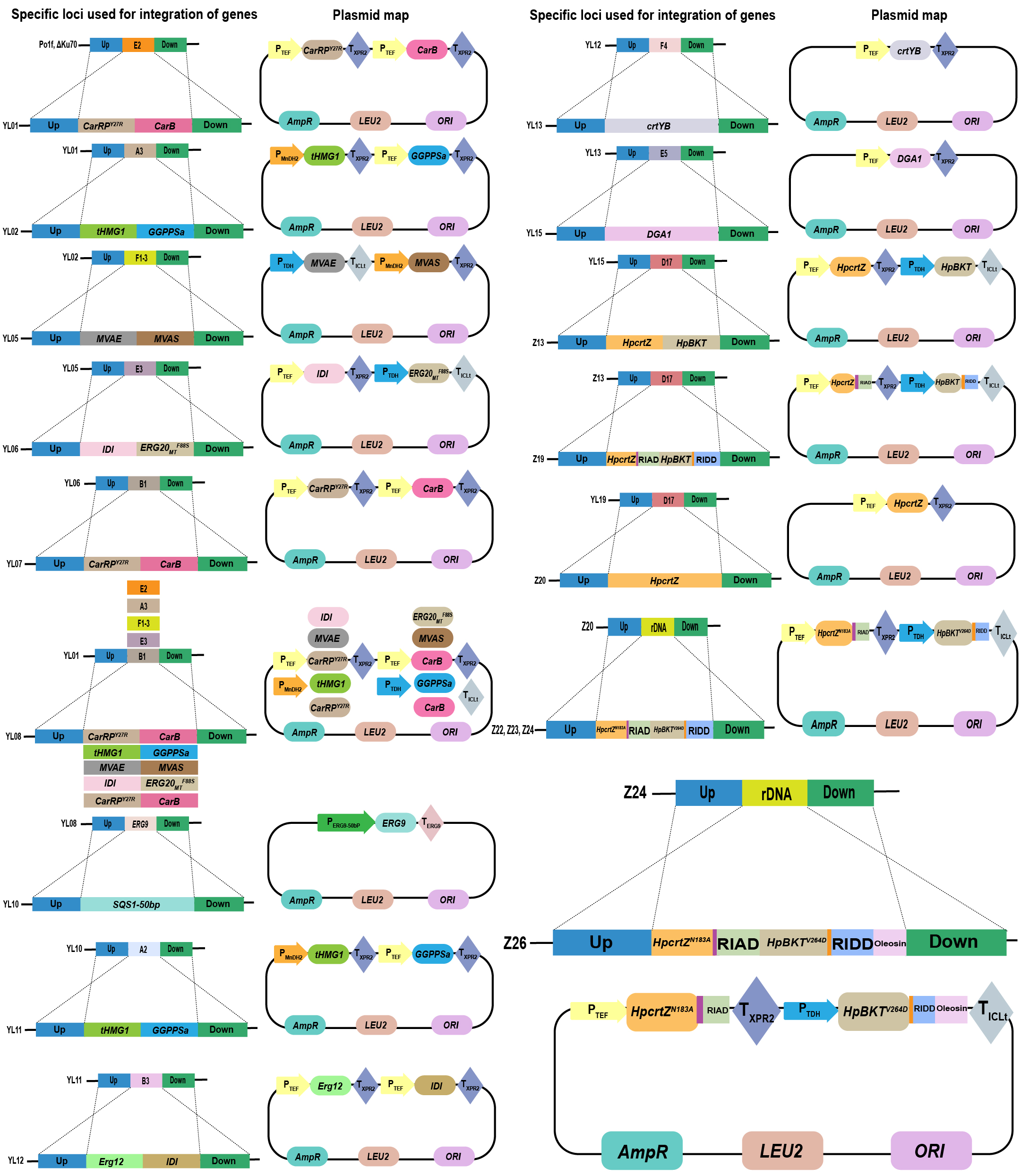
**

**Fig. S8. Schematic representation of genetic constructs used to construct strain Z26.**

**
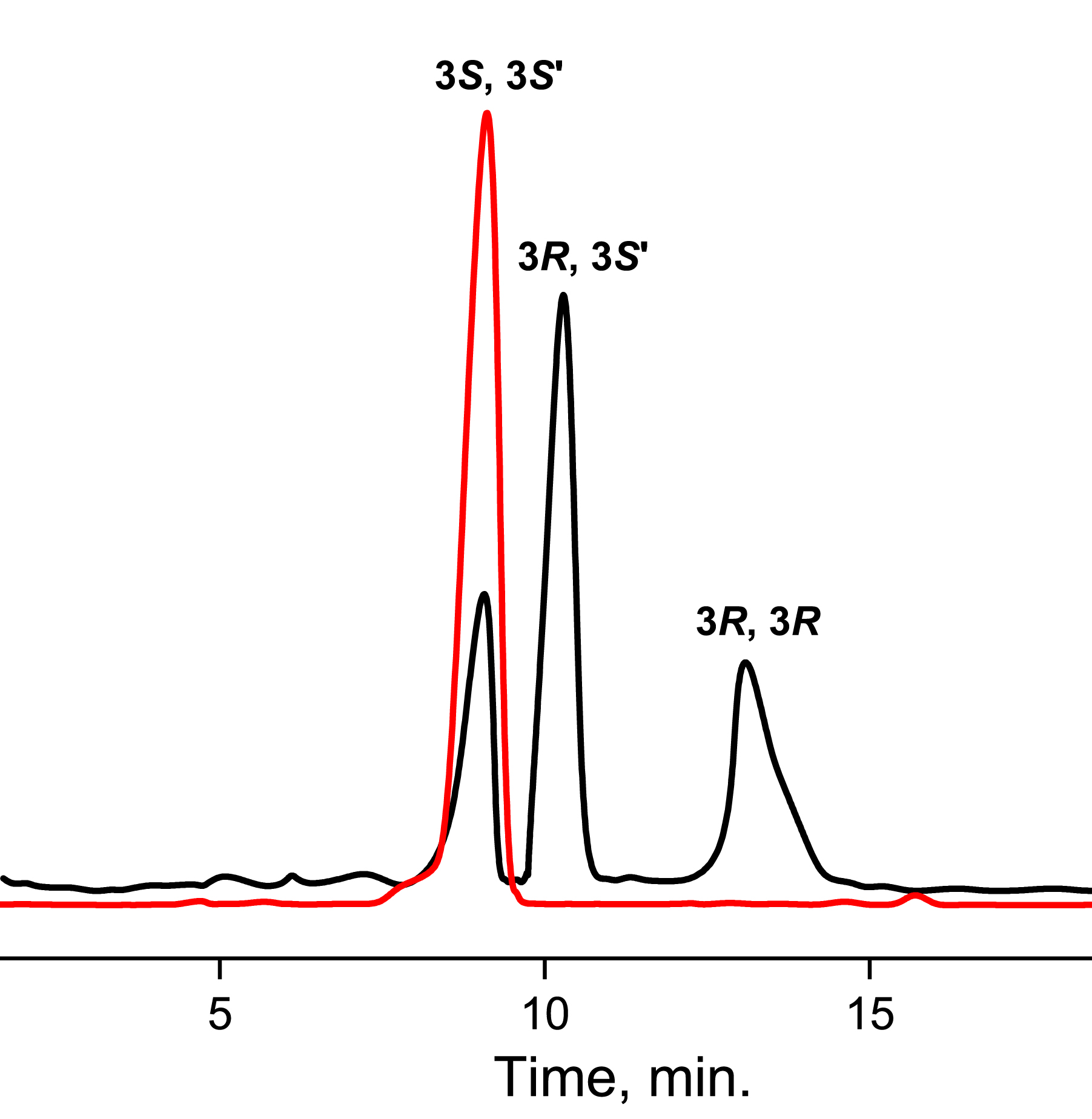
**

**Fig. S9.** **Chiral HPLC analysis of the astaxanthin generated by strain Z26.**

The black spectrum represents synthesized astaxanthin which composed of a mixture of (3*S*,3'*S*), (3*R*,3'*S*), and (3*R*,3'*R*) enantiomers, while the red spectrum represents astaxanthin generated by strain Z26.

**
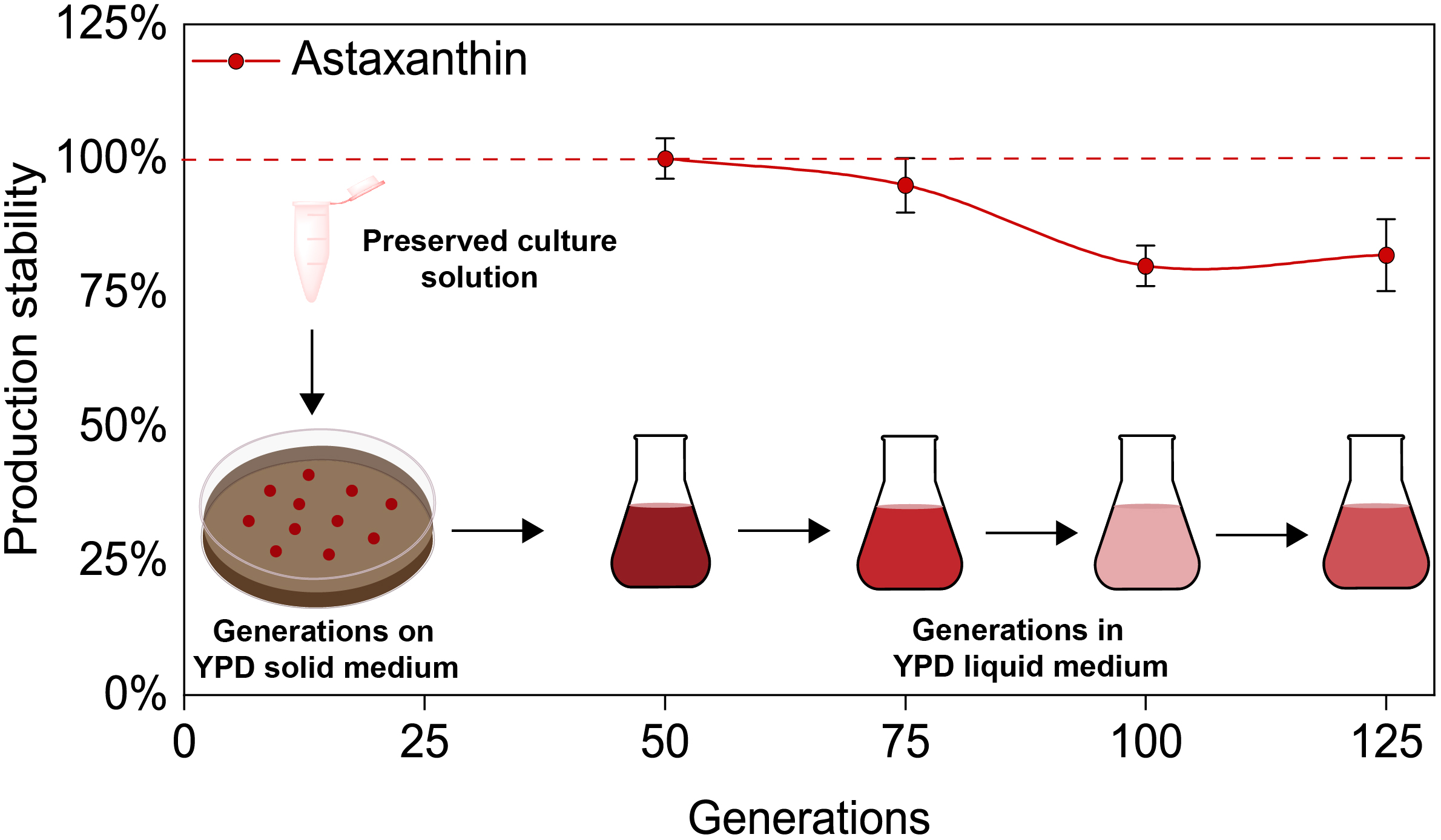
Fig. S10. Illustration of strain Z26 production stability according to the number of generations obtained in YPD medium.**

We calculated 25 generations for the modified strain to form colonies on YPD plates based on the mass of a yeast cell [1]. The constructed strain was put on YPD plate from the preserved culture solution then transferred to the liquid media.

**Supplementary Tables**

**Table S1. Plasmids used in this study.**

| Plasmids | Description | Sources |
| --- | --- | --- |
| pYLXP'1 | *Amp*,*Leu2* marker,TEF promoter and XPR2 terminator | Lab stock |
| pYLXP'2 | *Amp*,*Leu2* marker,MnDH2 promoter and XPR2 terminator | Lab stock |
| pYLXP'3 | *Amp*,*Leu2* marker,TDH promoter and ICLt terminator | This study |
| pYLXP'4 | Amp,*Ura3* marker,TEF promoter and XPR2 terminator | This study |
| pYLXP'5 | Amp,*Ura3* marker, MnDH2 promoter and XPR2 terminator | This study |
| pYL01 | pYLXP'1,E2-*P_TEF_-CarRP^Y27R^-T_XPR2_-P_TEF_-CarB-T_XPR2_* | This study |
| pYL02 | pYLXP'1'2,A3-*P_MNDH2_-tHMG1-T_XPR2_- P_TEF_ -GGPPSa-T_XPR2_* | This study |
| pYL03 | pYLXP'1'2,A3-*P_MNDH2_-tHMG1-T_XPR2_- P_TEF_ -GGPPXd-T_XPR2_* | This study |
| pYL04 | pYLXP'1'2,A3-*P_MNDH2_-tHMG1-T_XPR2_- P_TEF_ -GGPPYl-T_XPR2_* | This study |
| pYL05 | pYLXP'2'3,F1-3-*P_TDH_-MVAE-T_ICLt_- P_MNDH2_ -MVAS- T_XPR2_* | This study |
| pYL06 | pYLXP'1'3,E3-*P_TEF_-IDI-T_XPR2_- P_TDH_ –ERG20_MT_^F88S^- T_ICLt_* | This study |
| pYL07 | pYLXP'1,B1-*P_TEF_-CarRP^Y27R^-T_XPR2_-P_TEF_-CarB-T_XPR_* | This study |
| pYL09 | pYLXP'1,E13-*P_TEF_-CarRP^Y27R^-T_XPR2_-P_TEF_-CarB-T_XPR_* | This study |
| pYL10 | pYLXP'1, SQS1_-50_bp | This study |
| pYL11 | pYLXP'1'2,A2-*P_MNDH2_-tHMG1-T_XPR2_- P_TEF_ -GGPPSa-T_XPR2_* | This study |
| pYL12 | pYLXP'1,B3-*P_TEF_-Erg12-T_XPR2_-P_TEF_-IDI-T_XPR2_* | This study |
| pYL13 | pYLXP'1,F4-*P_TEF_-crtYB-T_XPR2_* | This study |
| pYL14 | pYLXP'1,E5-*P_TEF_-ACC1-T_XPR2_* | This study |
| pYL15 | pYLXP'1,E5-*P_TEF_-DGA1-T_XPR2_* | This study |
| pYL16 | pYLXP'1,A1-*P_TEF_-ZWF1-T_XPR2_-P_TEF_-GND1-T_XPR2_* | This study |
| pYL17 | pYLXP'1,F17- *P_TEF_-GND1-T_XPR2_* | This study |
| pYL18 | pYLXP'1'3,F1::*P_TEF_-CarRP^Y27R^*-RIAD*-T_XPR2_-P_TDH_–*ERG20_MT_^F88S^*-* RIDD-*T_ICLt_* | This study |
| pYL19 | pYLXP'1'3,F1::*P_TEF_-CarRP^Y27R^*-RIDD*-T_XPR2_-P_TDH_–*ERG20_MT_^F88S^*-* RIAD-*T_ICLt_* | This study |
| pYL20 | pYLXP'1'3,AXP:: *P_TEF_-IDI*-RIAD*-T_XPR2_-P_TDH_–*GGPPSa*-* RIDD-*T_ICLt_* | This study |
| pZ01-Z18 | pYLXP'4,D17::*P_TEF_ -HpcrtZ-T_XPR2-_P_TDH_-HpBKT-T_ICLt_* | This study |
| pZ19 | pYLXP'4,D17::*P_TEF_-HpcrtZ-*RIAD*-T_XPR2-_P_TDH_-HpBKT-*RIDD*-T_ICLt_* | This study |
| pZ20 | pYLXP'4,rDNA::*P_TEF_ -HpcrtZ-T_XPR2_* | This study |
| pZ21 | pYLXP'5,rDNA:: *P_TEF_-HpBKT-T_ICLt_* | This study |
| pZ22 | pYLXP'4,rDNA::*P_TEF_-HpcrtZ^N183A^-*RIAD*-T_XPR2-_P_TDH_-HpBKT^V264D^ -*RIDD*-T_ICLt_* | This study |
| pZ23 | pYLXP'4,rDNA::*P_TEF_-HpcrtZ^N183A^-*RIAD*-T_XPR2-_P_TDH_-HpBKT^V264D^ -*RIDD*-T_ICLt_* | This study |
| pZ24 | pYLXP'4,rDNA::*P_TEF_-HpcrtZ^N183A^-*RIAD*-T_XPR2-_P_TDH_-HpBKT^V264D^ -*RIDD*-T_ICLt_* | This study |
| pZ25 | pYLXP'4,rDNA::*P_TEF_-HpcrtZ^N183A^-*RIAD*-T_XPR2-_P_TDH_-HpBKT^V264D^*-RIDD-KDEL*-T_ICLt_* | This study |
| pZ26 | pYLXP'4,rDNA::*P_TEF_-HpcrtZ^N183A^-*RIAD*-T_XPR2-_P_TDH_-HpBKT^V264D^*-RIDD-Oleosin*-T_ICLt_* | This study |
| pZ27 | pYLXP'4,rDNA::*P_TEF_-HpcrtZ^N183A^-*RIAD*-T_XPR2-_P_TDH_-HpBKT^V264D^-*RIDD*-*SKL*-T_ICLt_* | This study |
| pBKT01 | pYLXP'3,D17:: *P_TDH_-HpBKT* ^M61A^*-T_ICLt_* | This study |
| pBKT02 | pYLXP'3,D17:: *P_TDH_-HpBKT* ^T64A^*-T_ICLt_* | This study |
| pBKT03 | pYLXP'3,D17:: *P_TDH_-HpBKT* ^I65A^*-T_ICLt_* | This study |
| pBKT04 | pYLXP'3,D17:: *P_TDH_-HpBKT* ^T68A^*-T_ICLt_* | This study |
| pBKT05 | pYLXP'3,D17:: *P_TDH_-HpBKT* ^I116A^*-T_ICLt_* | This study |
| pBKT06 | pYLXP'3,D17:: *P_TDH_-HpBKT* ^V117A^*-T_ICLt_* | This study |
| pBKT07 | pYLXP'3,D17:: *P_TDH_-HpBKT* ^V117A^*-T_ICLt_* | This study |
| pBKT08 | pYLXP'3,D17:: *P_TDH_-HpBKT* ^E119A^*-T_ICLt_* | This study |
| pBKT09 | pYLXP'3,D17:: *P_TDH_-HpBKT* ^F120A^*-T_ICLt_* | This study |
| pBKT10 | pYLXP'3,D17:: *P_TDH_-HpBKT* ^L121A^*-T_ICLt_* | This study |
| pBKT11 | pYLXP'3,D17:: *P_TDH_-HpBKT* ^L238A^*-T_ICLt_* | This study |
| pBKT12 | pYLXP'3,D17:: *P_TDH_-HpBKT* ^F241A^*-T_ICLt_* | This study |
| pBKT13 | pYLXP'3,D17:: *P_TDH_-HpBKT* ^R242A^*-T_ICLt_* | This study |
| pBKT14 | pYLXP'3,D17:: *P_TDH_-HpBKT* ^F246A^*-T_ICLt_* | This study |
| pBKT15 | pYLXP'3,D17:: *P_TDH_-HpBKT* ^F281A^*-T_ICLt_* | This study |
| pBKT16 | pYLXP'3,D17:: *P_TDH_-HpBKT* ^L282A^*-T_ICLt_* | This study |
| pBKT17 | pYLXP'3,D17:: *P_TDH_-HpBKT* ^T283A^*-T_ICLt_* | This study |
| pBKT18 | pYLXP'3,D17:: *P_TDH_-HpBKT* ^Y285A^*-T_ICLt_* | This study |
| pBKT19 | pYLXP'3,D17:: *P_TDH_-HpBKT* ^W301A^*-T_ICLt_* | This study |
| pBKT20 | pYLXP'3,D17:: *P_TDH_-HpBKT* ^I65F^*-T_ICLt_* | This study |
| pBKT21 | pYLXP'3,D17:: *P_TDH_-HpBKT* ^I116F^*-T_ICLt_* | This study |
| pBKT22 | pYLXP'3,D17:: *P_TDH_-HpBKT* ^V117F^*-T_ICLt_* | This study |
| pBKT23 | pYLXP'3,D17:: *P_TDH_-HpBKT* ^E119D^*-T_ICLt_* | This study |
| pBKT24 | pYLXP'3,D17:: *P_TDH_-HpBKT* ^F120W^*-T_ICLt_* | This study |
| pBKT25 | pYLXP'3,D17:: *P_TDH_-HpBKT* ^F120Y^*-T_ICLt_* | This study |
| pBKT26 | pYLXP'3,D17:: *P_TDH_-HpBKT* ^L121F^*-T_ICLt_* | This study |
| pBKT27 | pYLXP'3,D17:: *P_TDH_-HpBKT* ^L238F^*-T_ICLt_* | This study |
| pBKT28 | pYLXP'3,D17:: *P_TDH_-HpBKT* ^F241W^*-T_ICLt_* | This study |
| pBKT29 | pYLXP'3,D17:: *P_TDH_-HpBKT* ^F241Y^*-T_ICLt_* | This study |
| pBKT30 | pYLXP'3,D17:: *P_TDH_-HpBKT* ^F246Y^*-T_ICLt_* | This study |
| pBKT31 | pYLXP'3,D17:: *P_TDH_-HpBKT* ^F246W^*-T_ICLt_* | This study |
| pBKT32 | pYLXP'3,D17:: *P_TDH_-HpBKT* ^L282F^*-T_ICLt_* | This study |
| pBKT33 | pYLXP'3,D17:: *P_TDH_-HpBKT* ^W301F^*-T_ICLt_* | This study |
| pBKT34 | pYLXP'3,D17:: *P_TDH_-HpBKT* ^W301Y^*-T_ICLt_* | This study |
| pBKT35 | pYLXP'3,D17:: *P_TDH_-HpBKT* ^F298Y^*-T_ICLt_* | This study |
| pBKT36 | pYLXP'3,D17:: *P_TDH_-HpBKT* ^H165R^*-T_ICLt_* | This study |
| pBKT37 | pYLXP'3,D17:: *P_TDH_-HpBKT* ^V264D^*-T_ICLt_* | This study |
| pBKT38 | pYLXP'3,D17:: *P_TDH_-HpBKT* ^R242A^*-T_ICLt_* | This study |
| pBKT39 | pYLXP'3,D17:: *P_TDH_-HpBKT* ^R242C^*-T_ICLt_* | This study |
| pBKT40 | pYLXP'3,D17:: *P_TDH_-HpBKT* ^R242D^*-T_ICLt_* | This study |
| pBKT41 | pYLXP'3,D17:: *P_TDH_-HpBKT* ^R242E^*-T_ICLt_* | This study |
| pBKT42 | pYLXP'3,D17:: *P_TDH_-HpBKT* ^R242F^*-T_ICLt_* | This study |
| pBKT43 | pYLXP'3,D17:: *P_TDH_-HpBKT* ^R242G^*-T_ICLt_* | This study |
| pBKT44 | pYLXP'3,D17:: *P_TDH_-HpBKT* ^R242H^*-T_ICLt_* | This study |
| pBKT45 | pYLXP'3,D17:: *P_TDH_-HpBKT* ^R242I^*-T_ICLt_* | This study |
| pBKT46 | pYLXP'3,D17:: *P_TDH_-HpBKT* ^R242K^*-T_ICLt_* | This study |
| pBKT47 | pYLXP'3,D17:: *P_TDH_-HpBKT* ^R242L^*-T_ICLt_* | This study |
| pBKT48 | pYLXP'3,D17:: *P_TDH_-HpBKT* ^R242M^*-T_ICLt_* | This study |
| pBKT49 | pYLXP'3,D17:: *P_TDH_-HpBKT* ^R242N^*-T_ICLt_* | This study |
| pBKT50 | pYLXP'3,D17:: *P_TDH_-HpBKT* ^R242P^*-T_ICLt_* | This study |
| pBKT51 | pYLXP'3,D17:: *P_TDH_-HpBKT* ^R242Q^*-T_ICLt_* | This study |
| pBKT52 | pYLXP'3,D17:: *P_TDH_-HpBKT* ^R242S^*-T_ICLt_* | This study |
| pBKT53 | pYLXP'3,D17:: *P_TDH_-HpBKT* ^R242T^*-T_ICLt_* | This study |
| pBKT54 | pYLXP'3,D17:: *P_TDH_-HpBKT* ^R242V^*-T_ICLt_* | This study |
| pBKT55 | pYLXP'3,D17:: *P_TDH_-HpBKT* ^R242W^*-T_ICLt_* | This study |
| pBKT56 | pYLXP'3,D17:: *P_TDH_-HpBKT* ^R242Y^*-T_ICLt_* | This study |
| pBKT57 | pYLXP'3,D17:: *P_TDH_-HpBKT* ^F246C^*-T_ICLt_* | This study |
| pBKT58 | pYLXP'3,D17:: *P_TDH_-HpBKT* ^F246D^*-T_ICLt_* | This study |
| pBKT59 | pYLXP'3,D17:: *P_TDH_-HpBKT* ^F246E^*-T_ICLt_* | This study |
| pBKT60 | pYLXP'3,D17:: *P_TDH_-HpBKT* ^F246G^*-T_ICLt_* | This study |
| pBKT61 | pYLXP'3,D17:: *P_TDH_-HpBKT* ^F246H^*-T_ICLt_* | This study |
| pBKT62 | pYLXP'3,D17:: *P_TDH_-HpBKT* ^F246I^*-T_ICLt_* | This study |
| pBKT63 | pYLXP'3,D17:: *P_TDH_-HpBKT* ^F246K^*-T_ICLt_* | This study |
| pBKT64 | pYLXP'3,D17:: *P_TDH_-HpBKT* ^F246L^*-T_ICLt_* | This study |
| pBKT65 | pYLXP'3,D17:: *P_TDH_-HpBKT* ^F246M^*-T_ICLt_* | This study |
| pBKT66 | pYLXP'3,D17:: *P_TDH_-HpBKT* ^F246N^*-T_ICLt_* | This study |
| pBKT67 | pYLXP'3,D17:: *P_TDH_-HpBKT* ^F246P^*-T_ICLt_* | This study |
| pBKT68 | pYLXP'3,D17:: *P_TDH_-HpBKT* ^F246Q^*-T_ICLt_* | This study |
| pBKT69 | pYLXP'3,D17:: *P_TDH_-HpBKT* ^F246R^*-T_ICLt_* | This study |
| pBKT70 | pYLXP'3,D17:: *P_TDH_-HpBKT* ^F246S^*-T_ICLt_* | This study |
| pBKT71 | pYLXP'3,D17:: *P_TDH_-HpBKT* ^F246T^*-T_ICLt_* | This study |
| pBKT72 | pYLXP'3,D17:: *P_TDH_-HpBKT* ^F246V^*-T_ICLt_* | This study |
| pBKT73 | pYLXP'3,D17:: *P_TDH_-HpBKT* ^A157R^*-T_ICLt_* | This study |
| pBKT74 | pYLXP'3,D17:: *P_TDH_-HpBKT* ^A215T^*-T_ICLt_* | This study |
| pBKT75 | pYLXP'3,D17:: *P_TDH_-HpBKT* ^A215W^*-T_ICLt_* | This study |
| pBKT76 | pYLXP'3,D17:: *P_TDH_-HpBKT* ^A240M^*-T_ICLt_* | This study |
| pBKT77 | pYLXP'3,D17:: *P_TDH_-HpBKT* ^D183N^*-T_ICLt_* | This study |
| pBKT78 | pYLXP'3,D17:: *P_TDH_-HpBKT* ^F126H^*-T_ICLt_* | This study |
| pBKT79 | pYLXP'3,D17:: *P_TDH_-HpBKT* ^F184Y^*-T_ICLt_* | This study |
| pBKT80 | pYLXP'3,D17:: *P_TDH_-HpBKT* ^F198H^*-T_ICLt_* | This study |
| pBKT81 | pYLXP'3,D17:: *P_TDH_-HpBKT* ^F244G^*-T_ICLt_* | This study |
| pBKT82 | pYLXP'3,D17:: *P_TDH_-HpBKT* ^H171K^*-T_ICLt_* | This study |
| pBKT83 | pYLXP'3,D17:: *P_TDH_-HpBKT* ^H185L^*-T_ICLt_* | This study |
| pBKT84 | pYLXP'3,D17:: *P_TDH_-HpBKT* ^P182Y^*-T_ICLt_* | This study |
| pBKT85 | pYLXP'3,D17:: *P_TDH_-HpBKT* ^S197Y^*-T_ICLt_* | This study |
| pBKT86 | pYLXP'3,D17:: *P_TDH_-HpBKT* ^W194A^*-T_ICLt_* | This study |
| pBKT87 | pYLXP'3,D17:: *P_TDH_-HpBKT* ^W194G^*-T_ICLt_* | This study |
| pBKT88 | pYLXP'3,D17:: *P_TDH_-HpBKT* ^W194L^*-T_ICLt_* | This study |
| pBKT89 | pYLXP'3,D17:: *P_TDH_-HpBKT* ^W194P^*-T_ICLt_* | This study |
| pBKT90 | pYLXP'3,D17:: *P_TDH_-HpBKT* ^W194R^*-T_ICLt_* | This study |
| pBKT91 | pYLXP'3,D17:: *P_TDH_-HpBKT* ^W194S^*-T_ICLt_* | This study |
| pCrtZ01 | pYLXP'1,D17::*P_TEF_ -HpcrtZ* ^I104A^*-T_XPR2_* | This study |
| pCrtZ02 | pYLXP'1,D17::*P_TEF_ -HpcrtZ* ^T107A^*-T_XPR2_* | This study |
| pCrtZ03 | pYLXP'1,D17::*P_TEF_ -HpcrtZ* ^T129A^*-T_XPR2_* | This study |
| pCrtZ04 | pYLXP'1,D17::*P_TEF_ -HpcrtZ* ^L130A^*-T_XPR2_* | This study |
| pCrtZ05 | pYLXP'1,D17::*P_TEF_ -HpcrtZ* ^V133A^*-T_XPR2_* | This study |
| pCrtZ06 | pYLXP'1,D17::*P_TEF_ -HpcrtZ* ^V134A^*-T_XPR2_* | This study |
| pCrtZ07 | pYLXP'1,D17::*P_TEF_ -HpcrtZ* ^L138A^*-T_XPR2_* | This study |
| pCrtZ08 | pYLXP'1,D17::*P_TEF_ -HpcrtZ* ^L210A^*-T_XPR2_* | This study |
| pCrtZ09 | pYLXP'1,D17::*P_TEF_ -HpcrtZ* ^G211A^*-T_XPR2_* | This study |
| pCrtZ10 | pYLXP'1,D17::*P_TEF_ -HpcrtZ* ^L214A^*-T_XPR2_* | This study |
| pCrtZ11 | pYLXP'1,D17::*P_TEF_ -HpcrtZ* ^Y241A^*-T_XPR2_* | This study |
| pCrtZ12 | pYLXP'1,D17::*P_TEF_ -HpcrtZ* ^M242A^*-T_XPR2_* | This study |
| pCrtZ13 | pYLXP'1,D17::*P_TEF_ -HpcrtZ* ^L245A^*-T_XPR2_* | This study |
| pCrtZ14 | pYLXP'1,D17::*P_TEF_ -HpcrtZ* ^F265A^*-T_XPR2_* | This study |
| pCrtZ15 | pYLXP'1,D17::*P_TEF_ -HpcrtZ* ^L266A^*-T_XPR2_* | This study |
| pCrtZ16 | pYLXP'1,D17::*P_TEF_ -HpcrtZ* ^N183A^*-T_XPR2_* | This study |
| pCrtZ17 | pYLXP'1,D17::*P_TEF_ -HpcrtZ* ^N183G^*-T_XPR2_* | This study |
| pCrtZ18 | pYLXP'1,D17::*P_TEF_ -HpcrtZ* ^N183H^*-T_XPR2_* | This study |
| pCrtZ19 | pYLXP'1,D17::*P_TEF_ -HpcrtZ* ^N183P^*-T_XPR2_* | This study |
| pCrtZ20 | pYLXP'1,D17::*P_TEF_ -HpcrtZ* ^Y219H^*-T_XPR2_* | This study |

**Table S2. Codon optimized nucleotide sequences of the heterologous genes used in this study**

| Gene | Sequence (5'-3') |
| --- | --- |
| *CarRP* (*Mucor circinelloides)* | ATGCTGCTGACCTACATGGAGGTCCACCTGTACTACACCCTGCCCGTCCTGGGCGTCCTGTCTTGGCTGTCCCGACCCTACTACACCGCCACCGACGCCCTGAAGTTCAAGTTCCTGACCCTGGTGGCCTTCACCACCGCCTCCGCTTGGGACAACTACATTGTCTACCACAAGGCCTGGTCCTACTGCCCCACCTGCGTGACCGCCGTCATTGGTTACGTGCCCCTGGAGGAGTACATGTTCTTCATCATTATGACCCTGCTGACCGTGGCCTTCACTAACCTGGTCATGCGATGGCACCTGCACTCTTTCTTCATCCGACCCGAGACCCCCGTGATGCAGTCTGTCCTGGTGCGACTGGTCCCCATCACCGCCCTGCTGATCACCGCCTACAAGGCCTGGCACCTGGCCGTCCCTGGTAAACCCCTGTTCTACGGCTCTTGCATTCTGTGGTACGCCTGCCCCGTGCTGGCCCTTCTGTGGTTCGGCGCTGGCGAGTACATGATGCGACGACCCCTGGCCGTCCTGGTGTCTATTGCCCTGCCCACCCTGTTCCTGTGCTGGGTCGACGTGGTCGCCATTGGCGCCGGAACCTGGGACATCTCCCTGGCTACCTCCACCGGCAAGTTCGTGGTGCCCCACCTGCCCGTGGAGGAGTTCATGTTCTTCGCCCTGATCAACACCGTGCTGGTGTTCGGTACCTGCGCCATCGACCGAACCATGGCCATTCTGCACCTGTTCAAGAACAAGTCCCCCTACCAGCGACCCTACCAGCACTCTAAGTCCTTCCTGCACCAGATCCTGGAGATGACCTGGGCCTTCTGTCTGCCCGACCAGGTCCTGCACTCTGACACCTTCCACGACCTGTCCGTCTCTTGGGACATCCTGCGAAAGGCCTCCAAGTCTTTCTACACCGCCTCTGCCGTCTTCCCCGGCGACGTTCGACAGGAGCTGGGTGTCCTGTACGCCTTCTGCCGAGCCACCGACGACCTGTGCGACAACGAGCAGGTGCCCGTCCAGACCCGAAAGGAGCAGCTGATCCTGACCCACCAGTTCGTCTCCGACCTGTTCGGCCAGAAGACCTCCGCCCCCACCGCTATTGACTGGGACTTCTACAACGACCAGCTGCCCGCCTCCTGCATTTCCGCCTTCAAGTCCTTCACCCGACTGCGACACGTCCTGGAGGCCGGAGCTATTAAGGAGCTGCTGGACGGTTACAAGTGGGACCTGGAGCGACGATCCATTCGAGACCAGGAGGACCTGCGATACTACTCCGCCTGCGTGGCCTCCTCTGTCGGCGAGATGTGCACCCGAATCATTCTGGCCCACGCCGACAAGCCCGCCTCCCGACAGCAGACTCAGTGGATCATCCAGCGAGCCCGAGAGATGGGTCTGGTCCTGCAGTACACCAACATCGCCCGAGACATTGTCACCGACTCCGAGGAGCTGGGCCGATGTTACCTGCCCCAGGACTGGCTGACCGAGAAGGAGGTGGCCCTGATCCAGGGCGGTCTGGCTCGAGAGATTGGCGAGGAGCGACTGCTGTCTCTGTCTCACCGACTGATCTACCAGGCCGACGAGCTGATGGTCGTCGCCAACAAGGGCATTGACAAGCTGCCCTCCCACTGCCAGGGTGGCGTGCGAGCTGCTTGCAACGTCTACGCCTCCATCGGCACCAAGCTGAAGTCCTACAAGCACCACTACCCCTCCCGAGCCCACGTGGGAAACTCTAAGCGAGTGGAGATCGCCCTGCTGTCTGTCTACAACCTGTACACCGCCCCCATTGCCACCTCTTCCACCACCCACTGTCGACAGGGTAAAATGCGAAACCTGAACACCATCTAA |
| *CarB*  (*M.circinelloides)* | ATGTCCAAGAAGCACATTGTGATCATTGGCGCCGGTGTCGGCGGCACCGCTACTGCTGCTCGACTGGCTCGAGAGGGCTTCAAGGTGACCGTGGTGGAGAAGAACGACTTCGGTGGTGGTCGATGCTCTCTGATTCACCACCAGGGCCACCGATTCGACCAGGGCCCTTCCCTGTACCTGATGCCCAAGTACTTCGAGGACGCCTTCGCCGACCTGGACGAGCGAATTCAGGACCACCTGGAGCTGCTGCGATGCGACAACAACTACAAGGTCCACTTCGACGACGGTGAGTCCATTCAGCTGTCCTCTGACCTGACCCGAATGAAGGCCGAGCTGGACCGAGTCGAGGGCCCTCTTGGCTTCGGCCGATTCCTGGACTTCATGAAGGAGACCCACATCCACTACGAGTCCGGTACCCTGATCGCCCTGAAGAAGAACTTCGAGTCTATCTGGGACCTGATCCGAATCAAGTACGCCCCCGAGATTTTCCGACTGCACCTGTTCGGCAAGATCTACGACCGAGCCTCCAAGTACTTCAAGACCAAGAAGATGCGAATGGCCTTCACCTTCCAGACCATGTACATGGGTATGTCTCCCTACGACGCCCCCGCCGTCTACTCTCTGCTGCAGTACACCGAGTTCGCCGAGGGCATCTGGTACCCCCGAGGTGGTTTCAACATGGTGGTCCAGAAGCTGGAGGCCATCGCCAAGCAGAAGTACGACGCCGAGTTCATCTATAACGCCCCCGTCGCCAAGATCAACACCGACGACGCCACCAAGCAGGTCACCGGTGTCACCCTGGAGAACGGTCACATTATCGACGCCGACGCCGTCGTCTGCAACGCCGATCTGGTCTACGCCTACCACAACCTGCTGCCCCCCTGTCGATGGACCCAGAACACCCTGGCCTCCAAGAAGCTGACCTCCTCCTCCATCTCTTTCTACTGGTCCATGTCCACCAAGGTCCCCCAGCTGGACGTCCACAACATTTTCCTGGCCGAGGCCTACCAGGAGTCCTTCGACGAGATCTTCAAGGACTTCGGTCTGCCCTCCGAGGCCTCCTTCTACGTCAACGTCCCCTCTCGAATCGACCCCTCCGCCGCTCCTGACGGAAAGGACTCTGTCATCGTCCTGGTGCCCATTGGCCACATGAAGTCTAAGACCGGTGACGCCTCTACCGAGAACTACCCCGCCATGGTCGACAAGGCCCGAAAGATGGTCCTGGCCGTGATTGAGCGACGACTGGGCATGTCCAACTTCGCCGACCTCATCGAGCACGAGCAGGTCAACGACCCCGCCGTGTGGCAGTCCAAGTTCAACCTGTGGCGAGGTTCTATTCTGGGTCTGTCTCACGACGTCCTGCAGGTGCTGTGGTTCCGACCCTCCACCAAGGACTCCACCGGCCGATACGACAACCTGTTCTTCGTGGGCGCCTCCACCCACCCCGGTACTGGTGTTCCCATCGTCCTGGCCGGCTCCAAGCTGACCTCTGACCAGGTGGTCAAGTCTTTCGGTAAAACCCCCAAGCCCCGAAAGATCGAGATGGAGAACACCCAGGCCCCCCTGGAGGAGCCTGACGCTGAGTCTACCTTCCCCGTCTGGTTCTGGCTGCGAGCCGCCTTCTGGGTCATGTTCATGTTCTTCTACTTCTTCCCCCAGTCTAACGGTCAGACCCCCGCCTCTTTCATCAACAACCTGCTGCCTGAGGTGTTCCGAGTCCACAACTCTAACGTCATCTAA |
| *GGPPxd*  *(X.*dendrorhous) | atgGACTACGCCAACATCCTGACCGCCATCCCCCTGGAGTTCACCCCCCAAGACGACATCGTGCTGCTGGAGCCCTACCACTACCTGGGCAAGAACCCCGGCAAGGAGATCCGATCTCAGCTGATCGAGGCCTTCAACTACTGGCTGGACGTGAAGAAGGAGGACCTGGAGGTGATTCAGAACGTGGTGGGCATGCTGCACACCGCCTCCCTGCTCATGGACGACGTGGAGGACTCTTCTGTGCTGCGACGAGGCTCTCCCGTGGCCCACCTGATCTACGGCATCCCTCAGACCATCAACACCGCCAACTACGTGTACTTCCTGGCCTACCAAGAGATCTTCAAGCTGCGACCCACCCCCATCCCCATGCCCGTGATCCCCCCCTCTTCTGCCTCTCTGCAATCTTCTGTGTCCTCTGCCTCTTCTTCCTCCTCCGCCTCTTCCGAGAACGGCGGCACCTCTACCCCCAACTCTCAGATCCCCTTCTCTAAGGACACCTACCTGGACAAGGTGATCACCGACGAGATGCTGTCTCTGCACCGAGGCCAAGGCCTGGAGCTGTTCTGGCGAGACTCTCTGACCTGTCCCTCTGAGGAAGAGTACGTGAAGATGGTGCTGGGCAAGACCGGCGGCCTGTTCCGAATCGCCGTGCGACTGATGATGGCCAAGTCTGAGTGTGACATCGACTTCGTGCAGCTGGTGAACCTGATCTCTATCTACTTTCAGATCCGAGACGACTACATGAACCTGCAATCTTCTGAGTACGCCCACAACAAGAACTTCGCCGAGGACCTGACCGAGGGCAAGTTCTCTTTCCCCACCATCCACTCTATCCACGCCAACCCCTCTTCTCGACTGGTGATCAACACCCTGCAGAAGAAGTCTACCTCTCCCGAGATCCTGCACCACTGTGTGAACTACATGCGAACCGAAACCCACTCTTTCGAGTACACCCAAGAGGTGCTGAACACCCTGTCTGGCGCCCTGGAGCGAGAGCTGGGCCGACTGCAAGGCGAGTTCGCCGAGGCCAACTCTAAGATCGACCTGGGCGACGTGGAGTCTGAGGGCCGAACCGGCAAGAACGTGAAGCTGGAGGCCATCCTGAAGAAGCTGGCCGACATCCCCCTGTAA |
| *HpCtrZ* (*Haematococcus pluvialis*) | ATGCTGTCGAAGCTGCAGTCAATCAGCGTCAAGGCCCGCCGCGTTGAACTAGCCCGCGACATCACGCGGCCCAAAGTCTGCCTGCATGCTCAGCGGTGCTCGTTAGTTCGGCTGCGAGTGGCAGCACCACAGACAGAGGAGGCGGTGGGAACCCAGCAGGCTGCCGGCGCGGGCGATGAGCACAGCGCCGATGTAGCACT CCAGCAGCTTGACCGGGCTATCGCAGAGCGTCGTGCCCGGCGCAAACGGGAGCAGCTGTCATACCAGGCTGCCGCCATTGCAGCATCAATTGGCGTGTCAGGCATTGCCATCTTCGCCACCTACCTGAGATTTGCCATGCACATGACCGTGGGCGGCGCAGTGCCATGGGGTGAAGTGGCTGGCACTCTCCTCTTGGTGGTTGGTGGCGCGCTCGGCATGGAGATGTATGCCCGCTATGCACACAAAGCCATCTGGCATGAGTCGCCTCTGGGCTGGCTGCTGCACAAGAGCCACCACACACCTCGCACTGGACCCTTTGAAGCCAACGACTTGTTTGCAATCATCAATGGACTGCCCGCCATGCTCCTGTGTACCTTTGGCTTCTGGCTGCCCAACGTCCTGGGGACGGCCTGCTTTGGCGCGGGGCTGGGCATCACGCTATACGGCATGGCATATATGTTTGTACACGATGGCCTGGTGCACAGGCGCTTTCCCACCGGGCCCATCGCTGGCCTGCCCTACATGAAGCGCCTGACAGTGGCCCACCAGCTACACCACAGCGGCAAGTACGGTGGCGCGCCCTGGGGCATGTTCTTGGGTCCACAGGAGCTGCAGCACATTCCAGGTGCGGCGGAGGAGGTGGAGCGACTGGTCCTGGAACTGGACTGGTCCAAGCGGTAG |
| *HpBKT* (*Haematococcus pluvialis)* | ATGCACGTCGCATCGGCACTAATGGTCGAGCAGAAAGGCAGTGAGGCAGCTGCTTCCAGCCCAGACGTCTTGAGAGCGTGGGCGACACAGTATCACATGCCATCCGAGTCGTCAGACGCAGCTCGTCCTGCGCTAAAGCACGCCTACAAACCTCCAGCATCTGACGCCAAGGGCATCACGATGGCGCTGACCATCATTGGCACCTGGACCGCAGTGTTTTTACACGCAATATTTCAAATCAGGCTACCGACATCCATGGACCAGCTTCACTGGTTGCCTGTGTCCGAAGCCACAGCCCAGCTTTTGGGCGGAAGCAGCAGCCTACTGCACATCGCTGCAGTCTTCATTGTACTTGAGTTCCTGTACACTGGTCTATTCATCACCACACATGACGCAATGCATGGCACCATAGCTTTGAGGCACAGGCAGCTCAATGATCTCCTTGGCAACATCTGCATATCACTGTACGCCTGGTTTGACTACAGCATGCTGCATCGCAAGCACTGGGAGCACCACAACCATACTGGCGAAGTGGGGAAAGACCCTGACTTCCACAAGGGAAATCCCGGCCTTGTCCCCTGGTTCGCCAGCTTCATGTCCAGCTACATGTCCCTGTGGCAGTTTGCCCGGCTGGCATGGTGGGCAGTGGTGATGCAAATGCTGGGGGCGCCCATGGCAAATCTCCTAGTCTTCATGGCTGCAGCCCCAATCTTGTCAGCATTCCGCCTCTTCTACTTCGGCACTTACCTGCCACACAAGCCTGAGCCAGGCCCTGCAGCAGGCTCTCAGGTGATGGCCTGGTTCAGGGCCAAGACAAGTGAGGCATCTGATGTGATGAGTTTCCTGACATGCTACCACTTTGACCTGCACTGGGAGCACCACAGGTGGCCCTTTGCCCCCTGGTGGCAGCTGCCCCACTGCCGCCGCCTGTCCGGGCGTGGCCTGGTGCCTGCCTTGGCATGA |
| *EfMVAE* (*Enterococcus faecalis)* | ATGAAGACCGTGGTGATCATCGACGCCCTGCGAACCCCCATCGGCAAGTACAAGGGCTCTCTGTCTCAAGTGTCTGCCGTGGACCTGGGCACCCACGTGACCACTCAGCTGCTGAAGCGACACTCTACCATCTCTGAGGAGATCGACCAAGTGATCTTCGGCAACGTGCTGCAAGCCGGCAACGGACAGAACCCCGCCCGACAGATCGCCATCAACTCTGGCCTGTCTCACGAGATCCCCGCCATGACCGTCAACGAGGTGTGTGGCTCTGGCATGAAGGCCGTGATCCTGGCCAAGCAGCTGATTCAGCTGGGCGAGGCCGAGGTGCTGATCGCCGGCGGCATCGAGAACATGTCTCAAGCCCCCAAGCTGCAGCGATTCAACTACGAAACCGAGTCTTACGACGCCCCCTTCTCTTCTATGATGTACGACGGCCTGACCGACGCCTTCTCTGGCCAAGCCATGGGCCTGACCGCCGAGAACGTGGCCGAGAAGTACCACGTGACCCGAGAGGAGCAAGATCAGTTCTCTGTGCACTCTCAACTGAAGGCCGCCCAAGCCCAAGCCGAGGGCATCTTCGCCGACGAGATCGCCCCCCTGGAGGTGTCTGGCACCCTGGTGGAGAAGGACGAGGGCATCCGACCCAACTCTTCTGTGGAGAAGCTGGGCACCCTGAAGACCGTGTTCAAGGAGGACGGCACCGTGACCGCCGGCAACGCCTCTACCATCAACGACGGCGCCTCTGCCCTGATCATCGCCTCTCAAGAGTACGCCGAGGCCCACGGCCTGCCCTACCTGGCCATCATCCGAGACTCTGTGGAGGTGGGCATCGACCCCGCCTACATGGGCATCTCTCCCATCAAGGCCATTCAGAAGCTGCTGGCCCGAAATCAGCTGACCACCGAGGAGATCGACCTGTACGAGATCAACGAGGCCTTCGCCGCCACCTCTATCGTGGTGCAGCGAGAGCTGGCCCTGCCCGAGGAGAAGGTGAACATCTACGGCGGAGGCATCTCTCTGGGCCACGCCATCGGCGCCACCGGCGCCCGACTGCTGACCTCTCTGTCTTATCAGCTGAATCAGAAGGAGAAGAAGTACGGCGTGGCCTCTCTGTGTATCGGCGGAGGCCTCGGACTGGCCATGCTGCTGGAGCGACCTCAGCAGAAGAAGAACTCTCGATTCTATCAGATGTCCCCCGAGGAACGACTGGCCTCTCTGCTGAACGAGGGACAGATCTCTGCCGACACCAAGAAGGAGTTCGAGAACACCGCCCTGTCTTCTCAGATCGCCAACCACATGATCGAGAATCAGATCTCTGAAACCGAGGTGCCCATGGGCGTGGGCCTGCACCTGACCGTGGACGAAACCGACTACCTGGTGCCCATGGCCACCGAGGAGCCCTCCGTGATCGCCGCCCTGTCTAACGGCGCCAAGATCGCCCAAGGCTTCAAGACCGTGAATCAGCAGCGACTGATGCGAGGACAGATCGTGTTCTACGACGTGGCCGACGCCGAGTCTCTGATCGACGAGCTGCAAGTGCGAGAAACCGAGATCTTTCAGCAAGCCGAGCTGTCTTACCCCTCTATCGTGAAGCGAGGAGGCGGCCTGCGAGATCTGCAGTACCGAGCCTTCGACGAGTCTTTCGTGTCTGTGGACTTCCTGGTGGACGTGAAGGACGCCATGGGCGCCAACATCGTGAACGCCATGCTGGAGGGCGTGGCCGAGCTGTTCCGAGAGTGGTTCGCCGAGCAGAAGATCCTGTTCTCTATCCTGTCTAACTACGCCACCGAGTCTGTGGTGACCATGAAGACCGCCATCCCCGTGTCTCGACTCTCTAAGGGCTCTAACGGCCGAGAGATCGCCGAGAAGATCGTGCTGGCCTCTCGATACGCCTCTCTGGACCCCTACCGAGCCGTGACCCACAACAAGGGCATCATGAACGGCATCGAGGCCGTGGTGCTGGCCACCGGCAACGACACCCGAGCCGTGTCTGCCTCTTGTCACGCCTTCGCCGTGAAGGAGGGCCGATACCAAGGCCTGACCTCTTG |
| *EfMVAS* (*Enterococcus faecalis)* | ATGACCATCGGCATCGACAAGATCTCTTTCTTCGTGCCCCCCTACTACATCGACATGACCGCCCTGGCCGAGGCCCGAAACGTGGACCCCGGCAAGTTCCACATCGGCATCGGCCAAGATCAGATGGCCGTGAACCCCATCTCTCAAGACATCGTGACCTTCGCCGCCAACGCCGCCGAGGCCATCCTGACCAAGGAGGACAAGGAGGCCATCGACATGGTGATCGTGGGCACCGAGTCTTCTATCGACGAGTCTAAGGCCGCTGCCGTGGTGCTGCACCGACTGATGGGCATTCAGCCCTTCGCCCGATCTTTCGAGATCAAGGAGGCCTGTTACGGCGCCACCGCCGGCCTGCAGCTGGCCAAGAACCACGTGGCCCTGCACCCCGACAAGAAGGTGCTGGTGGTGGCCGCCGACATCGCCAAGTACGGCCTCAACTCTGGCGGCGAGCCCACCCAAGGCGCCGGCGCCGTGGCCATGCTGGTGGCCTCTGAGCCCCGAATCCTCGCTCTGAAGGAGGACAACGTGATGCTGACCCAAGACATCTACGACTTCTGGCGACCCACCGGCCACCCCTACCCCATGGTGGACGGCCCCCTGTCTAACGAAACCTACATTCAGTCTTTCGCCCAAGTGTGGGACGAGCACAAGAAGCGAACCGGCCTGGACTTCGCCGACTACGACGCCCTGGCCTTCCACATCCCCTACACCAAGATGGGCAAGAAGGCCCTGCTGGCCAAGATCTCTGATCAGACCGAGGCCGAGCAAGAGCGAATCCTGGCTCGATATGAGGAGTCTATCATCTACTCTCGACGAGTGGGCAACCTGTACACCGGCTCTCTGTACCTGGGCCTGATCTCTCTGCTGGAGAACGCCACCACCCTGACCGCCGGCAATCAGATCGGCCTGTTCTCTTACGGCTCTGGCGCCGTGGCCGAGTTCTTCACCGGCGAGCTGGTGGCCGGCTATCAGAACCACCTGCAGAAGGAAACCCACCTGGCCCTGCTGGACAACCGAACCGAGCTGTCTATCGCCGAGTACGAGGCCATGTTCGCCGAAACCCTGGACACCGACATCGATCAGACCCTGGAGGACGAGCTGAAGTACTCTATCTCTGCCATCAACAACACCGTGCGATCTTACCGAAACTAA |
| *GGPPsa* (Sulfolobus *acidocaldarius)* | ATGTCTTACTTCGACAACTACTTCAACGAGATCGTGAACTCTGTGAACGACATCATCAAGTCTTATATCTCTGGCGACGTGCCCAAGCTGTACGAGGCCTCTTACCACCTGTTCACCTCTGGCGGCAAGCGACTGCGACCCCTGATCCTGACCATCTCTTCTGACCTGTTCGGCGGACAGCGAGAGCGAGCCTACTACGCCGGCGCCGCCATCGAGGTGCTGCACACCTTCACCCTGGTGCACGACGACATCATGGACCAAGACAACATCCGACGAGGCCTGCCCACTGTGCATGTCAAGTATGGCCTGCCTCTCGCCATCCTCGCCGGCGACCTGCTGCACGCCAAGGCCTTTCAGCTGCTGACCCAAGCCCTGAGAGGCCTGCCCTCCGAAACCATCATCAAGGCCTTCGACATCTTCACCCGATCTATCATTATCATCTCTGAGGGCCAAGCCGTGGACATGGAGTTCGAGGACCGAATCGACATCAAGGAGCAAGAGTACCTGGACATGATCTCTCGAAAGACCGCCGCCCTGTTCTCTGCCTCTTCCTCTATCGGCGCCCTGATCGCCGGCGCCAACGACAACGACGTGCGACTGATGTCTGACTTCGGCACCAACCTGGGCATCGCCTTTCAGATCGTGGACGACATCCTGGGCCTGACCGCCGACGAGAAGGAGCTGGGCAAGCCCGTGTTCTCTGACATCCGAGAGGGCAAAAAGACCATCCTGGTGATCAAGACCCTGGAGCTGTGTAAGGAGGACGAGAAGAAGATCGTGCTGAAGGCCCTGGGCAACAAGTCTGCCTCTAAGGAGGAGCTGATGTCCTCTGCCGACATTATCAAGAAGTATTCTCTGGACTACGCCTACAACCTGGCCGAGAAGTACTACAAGAACGCCATCGACTCTCTGAACCAAGTCTCTTCTAAGTCTGACATCCCCGGCAAGGCCCTGAAGTACCTGGCCGAGTTCACCATCCGAAGACGAAAGTAA |
| *PsCrtW*  (*Paracoccus sp.)* | ATGAGTGCCCACGCCCTGCCCAAGGCCGACCTGACCGCCACCTCCCTGATCGTGTCGGGTGGCATTATCGCCGCCTGGCTGGCCCTGCACGTCCACGCCCTCTGGTTCCTGGACGCTGCCGCCCACCCCATCCTGGCCATCGCCAACTTCCTGGGCCTGACCTGGCTGTCTGTCGGCCTGTTCATTATCGCCCACGACGCCATGCACGGCTCTGTCGTCCCCGGTAGACCCCGAGCTAACGCCGCCATGGGCCAGCTGGTCCTGTGGCTGTACGCCGGTTTCTCCTGGCGAAAGATGATCGTCAAGCACATGGCTCACCACCGACACGCCGGCACCGACGATGACCCCGACTTCGACCACGGCGGACCCGTCCGATGGTACGCCCGATTCATCGGCACCTACTTCGGTTGGCGAGAGGGCCTGCTGCTGCCCGTGATCGTCACCGTGTACGCCCTGATCCTGGGCGACCGATGGATGTACGTCGTCTTTTGGCCCCTGCCCTCCATATTAGCTAGTATCCAGTTGTTTGTATTCGGCACATGGCTGCCTCATAGACCAGGACATGATGCCTTCCCTGATAGGCATAACGCACGTAGTAGCCGAATTTCCGATCCCGTATCTCTCTTGACCTGCTTCCACTTTGGTGGTTACCATCATGAGCACCACCTCCACCCCACCGTCCCCTGGTGGCGACTTCCTTCCACCCGAACTAAGGGAGACACTGCT |
| *PACrtZ*  (*Pantoea Agglomerans)* | ATGCTGTGGATTTGGAACGCCCTGATTGTTCTGGTTACCGTTATTGGTATGGAGATTACTGCTGCTCTTGCTCACCGATACATTATGCATGGTTGGGGTTGGGGTTGGCATCTGTCTCACCACGAGCCTCACAAGGGATGGTTTGAGGTTAACGATCTTTACGCTGTTGTTTTCGCTGCTCTCTCTATTCTTCTCATCTACCTTGGTTCTACCGGAGTCTGGCCTCTGCAGTGGATCGGAGCTGGTATGACTCTTTACGGACTTCTTTACTTTATTGTTCATGACGGTCTCGTTCATCAGCGATGGCCTTTTCGATACGTTCCCCGACGAGGTTACCTCCGACGACTTTACATGGCTCATAGAATGCATCACGCTGTTAGAGGAAAGGAGGGTTGTGTTTCTTTTGGATTCCTGTACGCCCCTCCTCTTTCTAAGCTCCAGGCTACTCTTAGAGAGCGACACGGTGTTAAGCGAGGTGCTGCTCGAGACCAGCGATCTGTTGAGCGAGATGCCCCTCCCGGAAAG |
| Sequence of oleosin from *Zea mays* | ATGGCCGACCGAGACCGAAGCGGCATCTACGGCGGCGCCCACGCTACCTACGGCCAGCAGCAGCAGCAGGGCGGCGGTGGTCGACCTATGGGCGAGCAGGTCAAGGGCATGCTGCACGACAAGGGCCCCACTGCCTCCCAGGCCCTCACCGTTGCCACCCTGTTCCCCCTGGGCGGCCTCCTTCTCGTGCTTTCCGGTCTTGCCCTGACTGCCTCCGTGGTCGGCCTGGCTGTCGCCACTCCCGTCTTCCTCATCTTCTCCCCCGTCCTCGTCCCCGCCGCCCTTCTTATCGGCACCGCCGTCATGGGCTTCCTGACCTCTGGCGCCCTGGGCCTCGGCGGTCTTTCCTCTCTCACCTGCCTTGCCAACACCGCCCGACAGGCCTTTCAGCGAACCCCCGACTACGTCGAGGAGGCCCACCGACGAATGGCCGAGGCCGCTGCTCACGCCGGTCACAAGACCGCCCAGGCCGGACAGGCCATCCAGGGACGAGCCCAGGAGGCCGGTGCCGGTGGTGGTGCTGGTGCTGGAGCCGGAGGCGGTGGTCGAGCTTCCTCCTAA |
| Sequence of RIDD | GGGGGAGGTGGCTCTGGCGGCGGTGGTTCGGGCGGAGGAGGCTGTGGAAGCTTGAGAGAATGCGAGCTCTACGTGCAAAAACACAACATCCAGGCTCTTCTCAAGGACTCCATTGTCCAGCTGTGCACCGCACGTCCTGAACGGCCCATGGCCTTCCTGCGAGAGTACTTTGAGCGACTGGAGAAGGAGGAGGCCAAG |
| Sequence of RIAD | GGGGGTGGCGGCTCCGGCGGTGGAGGTTCTGGAGGAGGAGGCTGTGGTCTGGAGCAGTACGCCAACCAACTCGCAGACCAGATTATCAAGGAGGCTACCGAAGGCTGC |

**Table S3. Primers used in this study**

| Primers | Sequence (5'-3') |
| --- | --- |
| Crispr-AXP-F | GACCAGGTCGAAGTAGCTGGGTTTTAGAGCTAGAAATAGCAAGTTAAAATAAGGCT |
| Crispr-AXP-R | CTAAAACCCAGCTACTTCGACCTGGTCacgtcAACCTGCGCCGAC |
| Crispr-A3-F | ATACGAGATGAGTGCCAAAGGTTTTAGAGCTAGAAATAGCAAGTTAAAATAAGGCTAG |
| Crispr-A3-R | CTAAAACCTTTGGCACTCATCTCGTATacgtcAACCTGCGCCGACC |
| Crispr-D17-F | TCCGTAATATAGGTGACGACGTTTTAGAGCTAGAAATAGCAAGTTAAAATAAGGCTAG |
| Crispr-D17-R | CTAAAACGTCGTCACCTATATTACGGAacgtcAACCTGCGCCGAC |
| Crispr-F1-3-F | CTTGAGAGGAGCCAGGGGGAGTTTTAGAGCTAGAAATAGCAAGTTAAAATAAGGCTAG |
| Crispr-F1-3-R | CTAAAACTCCCCCTGGCTCCTCTCAAGacgtcAACCTGCGCCGAC |
| Crispr-E3-F | acagtaccagtacaatacacGTTTTAGAGCTAGAAATAGCAAGTTAAAATAAGGCTAG |
| Crispr-E3-R | CTAAAACgtgtattgtactggtactgtacgtcAACCTGCGCCGAC |
| Crispr-E5-F | acaagcatacagccctcgggGTTTTAGAGCTAGAAATAGCAAGTTAAAATAAGGCTAG |
| Crispr-E5-R | CTAAAACcccgagggctgtatgcttgtacgtcAACCTGCGCCG |
| Crispr-E2-F | TTTACTACCCACATCTGGGGGTTTTAGAGCTAGAAATAGCAAGTTAAAATAAGGCTAGTC |
| Crispr-E2-R | CTAAAACCCCCAGATGTGGGTAGTAAAacgtcAACCTGCGCCG |
| Crispr-F1-F | tcggcatctagcttgtttcgGTTTTAGAGCTAGAAATAGCAAGTTAAAATAAGGCTAGTCC |
| Crispr-F1-R | CTAAAACcgaaacaagctagatgccgaacgtcAACCTGCGCCG |
| Crispr-E13-F | TGGAGAACTGAGCTATACGAGTTTTAGAGCTAGAAATAGCAAGTTAAAATAAGGCTAGTC |
| Crispr-E13-R | CTAAAACTCGTATAGCTCAGTTCTCCAacgtcAACCTGCGCCGACC |
| Crispr-B1-F | GATGGTATTGTCCCGGTATCGTTTTAGAGCTAGAAATAGCAAGTTAAAATAAGGCTAGTC |
| Crispr-B1-R | CTAAAACGATACCGGGACAATACCATCacgtcAACCTGCGCCG |
| Crispr-A2-F | CGCAAACAGAACCGATGCGGGTTTTAGAGCTAGAAATAGCAAGTTAAAATAAGGCTAGTC |
| Crispr-A2-R | CTAAAACCCGCATCGGTTCTGTTTGCGacgtcAACCTGCGCCGAC |
| Crispr-F4-F | agttattgggaaacgtcgagGTTTTAGAGCTAGAAATAGCAAGTTAAAATAAGGCTAGTC |
| Crispr-F4-R | TAAAACctcgacgtttcccaataactacgtcAACCTGCGCCG |
| Crispr-A1-F | AGACGTGGCGAAACAACCAGGTTTTAGAGCTAGAAATAGCAAGTTAAAATAAGGCTAGTC |
| Crispr-A1-R | CTAAAACCTGGTTGTTTCGCCACGTCTacgtcAACCTGCGCCGACC |
| Crispr-F17-F | GCTTCGGTGGACACACACTGGTTTTAGAGCTAGAAATAGCAAGTTAAAATAAGGCTAGTC |
| Crispr-F17-R | CTAAAACCAGTGTGTGTCCACCGAAGCacgtcAACCTGCGCCGAC |
| Crispr-B3-F | AGCCGTCGGAACACCGCGGGGTTTTAGAGCTAGAAATAGCAAGTTAAAATAAGGCTAGTC |
| Crispr-B3-R | CTAAAACCCCGCGGTGTTCCGACGGCTacgtcAACCTGCGCCGACC |
| Crispr-MHY1-F | ggacgccgtttccatctcacGTTTTAGAGCTAGAAATAGCAAGTTAAAATAAGGCTAGTC |
| Crispr-MHY1-R | GTCGGCGCAGGTTgacgtggacgccgtttccatctcacGTTTTAG |
| Crispr-SQS1^50bp^-F | ttatttggcgtttagagttcGTTTTAGAGCTAGAAATAGCAAGTTAAAATAAGGCTAGTCCG |
| Crispr-SQS1^50bp^-R | GTCGGCGCAGGTTgacgtttatttggcgtttagagttcGTTTTAG |
| CarRP-F | actttttgcagtactaaccgcagCTGCTGACCTACATGGAGGTCC |
| CarRP-R | catagcacgcgtgtagatacTTAGATGGTGTTCAGGTTTCGCATTTTAC |
| CarB-F | cactttttgcagtactaaccgcagTCCAAGAAGCACATTGTGATCATTGGC |
| CarB-R | gatgcatagcacgcgtgtagatacTTAGATGACGTTAGAGTTGTGGACTCG |
| BKT-F | cagcactttttgcagtactaaccgcagCATGTCGCGAGTGCCCTCATG |
| BKT-R | ctcactcagatgcatagcacgcgtgtagatacCTACGCCAGCGCAGGCACCAATC |
| GGPPSa-F | ttttgcagtactaaccgcagTCTTACTTCGACAACTACTTCAACGAGATCG |
| GGPPSa-R | gcatagcacgcgtgtagatacTTACTTTCGTCTTCGGATGGTGAACT |
| IDI-F | cagcactttttgcagtactaaccgcagACGACGTCTTACAGCGACAAAATC |
| IDI-R | cagatgcatagcacgcgtgtagatacCTACTTGATCCACCGCCGAATCTC |
| GND1-F | ctttttgcagtactaaccgcagATGACTGACACTTCAAACATCAAGTGAG |
| GND1-R | catagcacgcgtgtagatacTTAAGCATCGTAAGTGGAAGAAGAAACCTC |
| CrtYB-F | ctttttgcagtactaaccgcagACCGCCCTGGCCTACTATCAG |
| CrtYB-R | catagcacgcgtgtagatacTTACTGTCCCTCCCAGCCAGAC |
| DGA1-F | ctttttgcagtactaaccgcagACTATCGACTCACAATACTACAAGTCGC |
| DGA1-R | catagcacgcgtgtagatacTTACTCAATCATTCGGAACTCTGGGGC |
| ACC1-F | ctttttgcagtactaaccgcagCGACTGCAATTGAGGACACTAACACG |
| ACC1-R | gcatagcacgcgtgtagatacTCACAACCCCTTGAGCAGCTC |
| GGPPXd-F | ctttttgcagtactaaccgcagGACTACGCCAACATCCTGACCG |
| GGPPXd-R | gcatagcacgcgtgtagatacTTACAGGGGGATGTCGGCC |
| MVAE-F | cttacacacaagacatatctacagcaATGAAGACCGTGGTGATCATCGAC |
| MVAE-R | cgttaaatatattttgctaaacaaactgcCAAGAGGTCAGGCCTTGGTATCG |
| ERG20-F | cacacaagacatatctacagcaATGTCCAAGGCGAAATTCGAAAGC |
| ERG20-R | gttaaatatattttgctaaacaaactgcCTACTTCTGTCGCTTGTAAATCTTGGC |
| GGPPYl-F | cttacacacaagacatatctacagcaATGGATTATAACAGCGCGGATTTCAAG |
| GGPPYl-R | cgttaaatatattttgctaaacaaactgcTCACTGCGCATCCTCAAAGTACTTTC |
| CrtZ-F | CCATACACAGCACCACCTCAATCATGCTCTCCAAGCTCCAGTCGATC |
| CrtZ-R | ctcagatgcatagcacgcgtgtagatacTCACCGTTTGGACCAGTCCAGC |
| MVAS-F | CATACACAGCACCACCTCAATCATGACCATCGGCATCGACAAGATC |
| MVAS-R | gcatagcacgcgtgtagatacTTAGTTTCGGTAAGATCGCACGGTGTTG |
| tHMG1-F | CATACACAGCACCACCTCAATCATGaccCAGTCTGTGAAGGTGGTTG |
| tHMG1-R | gatgcatagcacgcgtgtagatacCTATGACCGTATGCAAATATTCGAACCG |
| ZWF1-F | CCATACACAGCACCACCTCAATCATGACTGGCACCTTACCCAAGTTC |
| ZWF1-R | gatgcatagcacgcgtgtagatacTCACGAGGAGCCCTTGGTG |
| ERG12-F | CCATACACAGCACCACCTCAATCATGGACTACATCATTTCGGCGC |
| ERG12-R | gatgcatagcacgcgtgtagatacCTAATGGGTCCAGGGACCGATG |
| PsCrtW-F | cactttttgcagtactaaccgcagAGTGCCCACGCCCTGC |
| PsCrtW-R | gcatagcacgcgtgtagatacAGCAGTGTCTCCCTTAGTTCGG |
| PACrtZ-F | CATACACAGCACCACCTCAATCATGCTGTGGATTTGGAACGCC |
| PACrtZ-R | gcatagcacgcgtgtagatacCTTTCCGGGAGGGGCATCTC |
| CarRP-Y27R-F | GACCCcgaTACACCGCCACCGACGC |
| CarRP-Y27R-R | GGTGTAtcgGGGTCGGGACAGCCAAGACA |
| ERG20-F88S-F | GCGTTTAGTCTCGTGTCGGACGACATTATGG |
| ERG20-F88S-R | CACGAGACTAAACGCCTGCAGCAGCTC |
| M61A-F | GGGTATCACTGCCGCCTTGACCATCATTGGCACCTGGACCGCTGTCTTCC |
| M61A-R | CAAGGCGGCAGTGATACCCTTCGCATCGGAAGCAGGAGGCTTGTAGGCG |
| T64A-F | CTATGGCCTTGGCCATCATTGGCACCTGGACCGCTGTCTTCCTGCATGC |
| T64A-R | CAATGATGGCCAAGGCCATAGTGATACCCTTCGCATCGGAAGCAGGAGGCTTG |
| I65A-F | CCTTGACCGCCATTGGCACCTGGACCGCTGTCTTCCTGCATGCCATTTTCC |
| I65A-R | AGGTGCCAATGGCGGTCAAGGCCATAGTGATACCCTTCGCATCGGAAGCAGGAGG |
| T68A-F | CATCATTGGCGCTTGGACCGCTGTCTTCCTGCATGCCATTTTCCAGATTCGAC |
| T68A-R | GGTCCAAGCGCCAATGATGGTCAAGGCCATAGTGATACCCTTCGCATCGGAAGC |
| I116A-F | GCCGCTGTTTTCGCTGTCCTGGAGTTTCTCTACACAGGGTTATTCATCACCACCCACGACGC |
| I116A-R | CAGGACAGCGAAAACAGCGGCAATATGCAATAGCGACGAAGAACCTCCCAGCAGCTG |
| V117A-F | GCTGTTTTCATTGCTCTGGAGTTTCTCTACACAGGGTTATTCATCACCACCCACGAC |
| V117A-R | CTCCAGAGCAATGAAAACAGCGGCAATATGCAATAGCGACGAAGAACCTCCCAG |
| E119A-F | CATTGTCCTGGCTTTTCTCTACACAGGGTTATTCATCACCACCCACGACGC |
| E119A-R | GAGAAAAGCCAGGACAATGAAAACAGCGGCAATATGCAATAGCGACG |
| F120A-F | TGTCCTGGAGGCCCTCTACACAGGGTTATTCATCACCACCCACGACGCC |
| F120A-R | GTAGAGGGCCTCCAGGACAATGAAAACAGCGGCAATATGCAATAGCGACGAAGAAC |
| L121A-F | CTGGAGTTTGCCTACACAGGGTTATTCATCACCACCCACGACGCCATGC |
| L121A-R | CTGTGTAGGCAAACTCCAGGACAATGAAAACAGCGGCAATATGCAATAGCGACG |
| L238A-F | CCCCATCGCATCTGCATTTCGACTCTTCTACTTTGGAACGTATCTCCCCCATAAGCCCG |
| L238A-R | GAAATGCAGATGCGATGGGGGCAGCTGCCATAAACACCAGCAGGTTCGCC |
| F241A-F | CTCTGCAGCCCGACTCTTCTACTTTGGAACGTATCTCCCCCATAAGCCCGAG |
| F241A-R | AGAAGAGTCGGGCTGCAGAGAGGATGGGGGCAGCTGCCATAAACACCAG |
| R242A-F | CTCTCTGCATTTGCACTCTTCTACTTTGGAACGTATCTCCCCCATAAGCCCGAGC |
| R242A-R | GAAGAGTGCAAATGCAGAGAGGATGGGGGCAGCTGCCATAAACACC |
| F246A-F | GACTCTTCTACGCAGGAACGTATCTCCCCCATAAGCCCGAGCCTGGACCAG |
| F246A-R | CGTTCCTGCGTAGAAGAGTCGAAATGCAGAGAGGATGGGGGCAGCTGC |
| F281A-F | GTCATGTCGGCCCTGACTTGCTACCATTTTGACCTGCACTGGGAGCATCATCGGTG |
| F281A-R | CAAGTCAGGGCCGACATGACGTCGGAGGCCTCCGACGTTTTGGCTCGG |
| L282A-F | GTCGTTCGCCACTTGCTACCATTTTGACCTGCACTGGGAGCATCATCGGTGG |
| L282A-R | GGTAGCAAGTGGCGAACGACATGACGTCGGAGGCCTCCGACGTTTTGG |
| T283A-F | CGTTCCTGGCCTGCTACCATTTTGACCTGCACTGGGAGCATCATCGGTGG |
| T283A-R | ATGGTAGCAGGCCAGGAACGACATGACGTCGGAGGCCTCCGACGTTTTG |
| Y285A-F | CTGACTTGCGCCCATTTTGACCTGCACTGGGAGCATCATCGGTGGC |
| Y285A-R | CAAAATGGGCGCAAGTCAGGAACGACATGACGTCGGAGGCCTCCGACGTTTTG |
| W301A-F | CCCTGGGCACAACTTCCCCACTGTCGACGTCTTTCAGGAAGGGGATTGGTGCC |
| W301A-R | TGGGGAAGTTGTGCCCAGGGTGCGAAGGGCCACCGATGATGCTCCCA |
| I65F-F | CCTTGACCTTTATTGGCACCTGGACCGCTGTCTTCCTGCATGCCATTTTCC |
| I65F-R | AGGTGCCAATAAAGGTCAAGGCCATAGTGATACCCTTCGCATCGGAAGCAGGAGG |
| I116F-F | GCCGCTGTTTTCTTCGTCCTGGAGTTTCTCTACACAGGGTTATTCATCACCACCCACGACGC |
| I116F-R | CAGGACGAAGAAAACAGCGGCAATATGCAATAGCGACGAAGAACCTCCCAGCAGCTG |
| V117F-F | GCTGTTTTCATTTTCCTGGAGTTTCTCTACACAGGGTTATTCATCACCACCCACGAC |
| V117F-R | CTCCAGGAAAATGAAAACAGCGGCAATATGCAATAGCGACGAAGAACCTCCCAG |
| E119D-F | CATTGTCCTGGACTTTCTCTACACAGGGTTATTCATCACCACCCACGACGC |
| E119D-R | AGAGAAAGTCCAGGACAATGAAAACAGCGGCAATATGCAATAGCGACG |
| F120W-F | TGTCCTGGAGTACCTCTACACAGGGTTATTCATCACCACCCACGACGCC |
| F120W-R | GTAGAGGTACTCCAGGACAATGAAAACAGCGGCAATATGCAATAGCGACGAAGAAC |
| F120Y-F | TGTCCTGGAGTGGCTCTACACAGGGTTATTCATCACCACCCACGACGCC |
| F120Y-R | GTAGAGCCACTCCAGGACAATGAAAACAGCGGCAATATGCAATAGCGACGAAGAAC |
| L121F-F | CTGGAGTTTTTCTACACAGGGTTATTCATCACCACCCACGACGCCATGC |
| L121F-R | CTGTGTAGAAAAACTCCAGGACAATGAAAACAGCGGCAATATGCAATAGCGACG |
| L238F-F | CCCCATCTTCTCTGCATTTCGACTCTTCTACTTTGGAACGTATCTCCCCCATAAGCCCG |
| L238F-R | GAAATGCAGAGAAGATGGGGGCAGCTGCCATAAACACCAGCAGGTTCGCC |
| F241W-F | CTCTGCATGGCGACTCTTCTACTTTGGAACGTATCTCCCCCATAAGCCCGAG |
| F241W-R | AGAAGAGTCGCCATGCAGAGAGGATGGGGGCAGCTGCCATAAACACCAG |
| F241Y-F | CTCTGCATACCGACTCTTCTACTTTGGAACGTATCTCCCCCATAAGCCCGAG |
| F241Y-R | AGAAGAGTCGGTATGCAGAGAGGATGGGGGCAGCTGCCATAAACACCAG |
| F246W-F | GACTCTTCTACTGGGGAACGTATCTCCCCCATAAGCCCGAGCCTGGACCAG |
| F246W-R | CGTTCCCCAGTAGAAGAGTCGAAATGCAGAGAGGATGGGGGCAGCTGC |
| F246Y-F | GACTCTTCTACTATGGAACGTATCTCCCCCATAAGCCCGAGCCTGGACCAG |
| F246Y-R | CGTTCCATAGTAGAAGAGTCGAAATGCAGAGAGGATGGGGGCAGCTGC |
| L282F-F | GTCGTTCTTCACTTGCTACCATTTTGACCTGCACTGGGAGCATCATCGGTGG |
| L282F-R | GGTAGCAAGTGAAGAACGACATGACGTCGGAGGCCTCCGACGTTTTGG |
| W301F-F | CCCTGGTTCCAACTTCCCCACTGTCGACGTCTTTCAGGAAGGGGATTGGTGCC |
| W301F-R | TGGGGAAGTTGGAACCAGGGTGCGAAGGGCCACCGATGATGCTCCCA |
| W301Y-F | CCCTGGTACCAACTTCCCCACTGTCGACGTCTTTCAGGAAGGGGATTGGTGCC |
| W301Y-R | TGGGGAAGTTGGTACCAGGGTGCGAAGGGCCACCGATGATGCTCCCA |
| F298Y-F | GCCCTACGCACCCTGGTGGCAACTTCCCCACTGTCGACGTCTTTCAGG |
| F298Y-R | CCAGGGTGCGTAGGGCCACCGATGATGCTCCCAGTGCAGGTCAAAATGGTAGC |
| H165R-F | GTTCGACTACTCGATGCTCAGAAGAAAACACTGGGAACACCACAAC |
| H165R-R | GTGTTCCCAGTGTTTTCTTCTGAGCATCGAGTAGTCGAACCAGG |
| V264D-F | CAGCTGCCGGCTCCCAGGACATGGCTTGGTTCCGAGCCAAAACGTCGGAGG |
| V264D-R | CTCGGAACCAAGCCATGTCCTGGGAGCCGGCAGCTGGTCCAGG |
| R242A-F | CTGCATTTGCACTCTTCTACTTTGGAACGTATCTCCCCC |
| R242A-R | GTAGAAGAGTGCAAATGCAGAGAGGATGGGGGC |
| R242C-F | CTGCATTTTGCCTCTTCTACTTTGGAACGTATCTCCCCC |
| R242C-R | GTAGAAGAGGCAAAATGCAGAGAGGATGGGGGC |
| R242D-F | CTGCATTTGACCTCTTCTACTTTGGAACGTATCTCCCCC |
| R242D-R | GTAGAAGAGGTCAAATGCAGAGAGGATGGGGGC |
| R242E-F | CTGCATTTGAGCTCTTCTACTTTGGAACGTATCTCCCCC |
| R242E-R | GTAGAAGAGCTCAAATGCAGAGAGGATGGGGGC |
| R242F-F | CTGCATTTTTCCTCTTCTACTTTGGAACGTATCTCCCCC |
| R242F-R | GTAGAAGAGGAAAAATGCAGAGAGGATGGGGGC |
| R242G-F | CTGCATTTGGACTCTTCTACTTTGGAACGTATCTCCCCC |
| R242G-R | GTAGAAGAGTCCAAATGCAGAGAGGATGGGGGC |
| R242H-F | CTGCATTTCATCTCTTCTACTTTGGAACGTATCTCCCCC |
| R242H-R | GTAGAAGAGATGAAATGCAGAGAGGATGGGGGC |
| R242I-F | CTGCATTTATCCTCTTCTACTTTGGAACGTATCTCCCCC |
| R242I-R | GTAGAAGAGGATAAATGCAGAGAGGATGGGGGC |
| R242K-F | CTGCATTTAAGCTCTTCTACTTTGGAACGTATCTCCCCC |
| R242K-F | GTAGAAGAGCTTAAATGCAGAGAGGATGGGGGC |
| R242L-F | CTGCATTTCTCCTCTTCTACTTTGGAACGTATCTCCCCC |
| R242L-R | GTAGAAGAGGAGAAATGCAGAGAGGATGGGGGC |
| R242M-F | CTGCATTTATGCTCTTCTACTTTGGAACGTATCTCCCCC |
| R242M-R | GTAGAAGAGCATAAATGCAGAGAGGATGGGGGC |
| R242N-F | CTGCATTTAACCTCTTCTACTTTGGAACGTATCTCCCCC |
| R242N-R | GTAGAAGAGGTTAAATGCAGAGAGGATGGGGGC |
| R242P-F | CTGCATTTCCCCTCTTCTACTTTGGAACGTATCTCCCCC |
| R242P-R | GTAGAAGAGGGGAAATGCAGAGAGGATGGGGGC |
| R242Q-F | CTGCATTTCAGCTCTTCTACTTTGGAACGTATCTCCCCC |
| R242Q-R | GTAGAAGAGCTGAAATGCAGAGAGGATGGGGGC |
| R242S-F | CTGCATTTTCTCTCTTCTACTTTGGAACGTATCTCCCCC |
| R242S-R | GTAGAAGAGAGAAAATGCAGAGAGGATGGGGGC |
| R242T-F | CTGCATTTACGCTCTTCTACTTTGGAACGTATCTCCCCC |
| R242T-R | GTAGAAGAGCGTAAATGCAGAGAGGATGGGGGC |
| R242V-F | CTGCATTTGTGCTCTTCTACTTTGGAACGTATCTCCCCC |
| R242V-R | GTAGAAGAGCACAAATGCAGAGAGGATGGGGGC |
| R242W-F | CTGCATTTTGGCTCTTCTACTTTGGTACGTATCTCCCCC |
| R242W-R | GTAGAAGAGCCAAAATGCAGAGAGGATGGGGGC |
| R242Y-F | GCATTTTACCTCTTCTACTTTGGAACGTATCTCCCCC |
| R242Y-R | GTAGAAGAGGTAAAATGCAGAGAGGATGGGGGC |
| F246C-F | CTCTTCTACTGCGGAACGTATCTCCCCCATAAGCC |
| F246C-R | CGTTCCGCAGTAGAAGAGTCGAAATGCAGAGAGGATG |
| F246D-F | CTCTTCTACGACGGAACGTATCTCCCCCATAAGCC |
| F246D-R | CGTTCCGTCGTAGAAGAGTCGAAATGCAGAGAGGATG |
| F246E-F | CTTCTACGAGGGAACGTATCTCCCCCATAAGCC |
| F246E-R | CGTTCCCTCGTAGAAGAGTCGAAATGCAGAGAGGATG |
| F246G-F | CTTCTACGGAGGAACGTATCTCCCCCATAAGCC |
| F246G-R | CGTTCCTCCGTAGAAGAGTCGAAATGCAGAGAGGATG |
| F246H-F | CTTCTACCATGGAACGTATCTCCCCCATAAGCC |
| F246H-R | CGTTCCATGGTAGAAGAGTCGAAATGCAGAGAGGATG |
| F246I-F | CTTCTACATCGGAACGTATCTCCCCCATAAGCC |
| F246I-R | CGTTCCGATGTAGAAGAGTCGAAATGCAGAGAGGATG |
| F246K-F | CTTCTACAAGGGAACGTATCTCCCCCATAAGCC |
| F246K-R | CGTTCCCTTGTAGAAGAGTCGAAATGCAGAGAGGATG |
| F246L-F | CTCTGCACTCCGACTCTTCTACTTTGGAACGTATCTCC |
| F246L-R | GAGTCGGAGTGCAGAGAGGATGGGGGCAG |
| F246M-F | CTTCTACATGGGAACGTATCTCCCCCATAAGCC |
| F246M-R | CGTTCCCATGTAGAAGAGTCGAAATGCAGAGAGGATG |
| F246N-F | CTTCTACAACGGAACGTATCTCCCCCATAAGCC |
| F246N-R | CGTTCCGTTGTAGAAGAGTCGAAATGCAGAGAGGATG |
| F246P-F | CTTCTACCCCGGAACGTATCTCCCCCATAAGCC |
| F246P-R | CGTTCCGGGGTAGAAGAGTCGAAATGCAGAGAGGATG |
| F246Q-F | CTTCTACCAGGGAACGTATCTCCCCCATAAGCC |
| F246Q-R | CGTTCCCTGGTAGAAGAGTCGAAATGCAGAGAGGATG |
| F246R-F | CTTCTACCGGGGAACGTATCTCCCCCATAAGCC |
| F246R-R | CGTTCCCCGGTAGAAGAGTCGAAATGCAGAGAGGATG |
| F246S-F | CTTCTACTCTGGAACGTATCTCCCCCATAAGCC |
| F246S-R | CGTTCCAGAGTAGAAGAGTCGAAATGCAGAGAGGATG |
| F246T-F | CTTCTACACGGGAACGTATCTCCCCCATAAGCC |
| F246T-R | CGTTCCCGTGTAGAAGAGTCGAAATGCAGAGAGGATG |
| F246V-F | CTTCTACGTGGGAACGTATCTCCCCCATAAGCC |
| F246V-R | CGTTCCCACGTAGAAGAGTCGAAATGCAGAGAGGATG |
| I104A-F | CTGGTATTGCGGCCTTTGCCACCTACCTGAGATTCGCCATGCACATGAC |
| I104A-R | GGCAAAGGCCGCAATACCAGATACGCCAATTGAAGCAGCAATAGCC |
| T107A-F | GCGATCTTTGCCGCCTACCTGAGATTCGCCATGCACATGACCGTCGG |
| T107A-R | CAGGTAGGCGGCAAAGATCGCAATACCAGATACGCCAATTGAAGCAGC |
| T129A-F | CGAGGTGGCCGGAGCCCTACTTCTGGTTGTGGGCGGTGCCCTGG |
| T129A-R | GAAGTAGGGCTCCGGCCACCTCGCCCCAGG |
| L130A-F | GGTGGCCGGAACGGCCCTTCTGGTTGTGGGCGGTGCCCTGG |
| L130A-R | CCAGAAGGGCCGTTCCGGCCACCTCGCCCC |
| V133A-F | GAACGCTACTTCTGGCCGTGGGCGGTGCCCTGGGCATG |
| V133A-R | CCGCCCACGGCCAGAAGTAGCGTTCCGGCCACCTCGC |
| V134A-F | GCTACTTCTGGTTGCCGGCGGTGCCCTGGGCATGGAG |
| V134A-R | CACCGCCGGCAACCAGAAGTAGCGTTCCGGCCACCTC |
| L138A-F | GTGGGCGGTGCCGCCGGCATGGAGATGTACGCGAGATACGCCCATAAGG |
| L138A-R | CCATGCCGGCGGCACCGCCCACAACCAGAAGTAGCG |
| L210A-F | CTTCGGAGCTGGCGCCGGAATAACATTGTATGGTATGGCATACATGTTTG |
| L210A-R | GTTATTCCGGCGCCAGCTCCGAAGCAGGC |
| G211A-F | GGAGCTGGCCTCGCAATAACATTGTATGGTATGGCATACATGTTTGTG |
| G211A-R | CAATGTTATTGCGAGGCCAGCTCCGAAGCAG |
| L214A-F | CTCGGAATAACAGCATATGGTATGGCATACATGTTTGTGCATG |
| L214A-R | CATACCATATGCTGTTATTCCGAGGCCAGCTCC |
| Y241A-F | CGGGCTACCCGCCATGAAGCGCCTGACGGTTGCACACCAGC |
| Y241A-R | CGCTTCATGGCGGGTAGCCCGGCAATAGGGCCAGTTGG |
| M242A-F | CTACCCTACGCCAAGCGCCTGACGGTTGCACACCAGC |
| M242A-R | GCGCTTGGCGTAGGGTAGCCCGGCAATAGGGCCAGTTGG |
| L245A-F | CATGAAGCGCGCCACGGTTGCACACCAGCTGCACCACAG |
| L245A-R | GCAACCGTGGCGCGCTTCATGTAGGGTAGCCCGGCAATAGG |
| F265A-F | GTGGGGAATGGCTCTGGGTCCCCAGGAACTGCAGCACATTCC |
| F265A-R | GGACCCAGAGCCATTCCCCACGGGGCTCCTCCGTACTTG |
| L266A-F | GGGGAATGTTCGCAGGTCCCCAGGAACTGCAGCACATTCCGG |
| L266A-R | CTGGGGACCTGCGAACATTCCCCACGGGGCTCCTCCGT |
| N183A-F | GCTATCATCGCTGGTCTCCCTGCGATGCTCC |
| N183A-R | CAGGGAGACCAGCGATGATAGCAAACAGGTCGTTGGC |
| N183G-F | GCTATCATCGGTGGTCTCCCTGCGATGCTCC |
| N183G-R | CAGGGAGACCACCGATGATAGCAAACAGGTCGTTGGC |
| N183H-F | CTATCATCCACGGTCTCCCTGCGATGCTCC |
| N183H-R | CAGGGAGACCGTGGATGATAGCAAACAGGTCGTTGGC |
| N183P-F | GCTATCATCCCTGGTCTCCCTGCGATGCTCC |
| N183P-R | GGGAGACCAGGGATGATAGCAAACAGGTCGTTGGC |
| Y219H-F | GGTATGGCACATATGTTTGTGCATGATGGACTAGTCCACAG |
| Y219H-R | GCACAAACATATGTGCCATACCATACAATGTTATTCCGAGGC |
| A157R-F | CTCCCTTTATCGATGGTTCGACTACTCGATGCTCC |
| A157R-R | GTCGAACCATCGATAAAGGGAGATGCAGATATTGCCCAATAG |
| A215T-F | GCCTGGTGGACCGTGGTTATGCAGATGCTGGGTGC |
| A215T-R | GCATAACCACGGTCCACCAGGCCAGTCGGG |
| A215W-F | GCCTGGTGGTGGGTGGTTATGCAGATGCTGGGTGC |
| A215W-R | CATAACCACCCACCACCAGGCCAGTCGGG |
| A240M-F | CATCCTCTCTATGTTTCGACTCTTCTACTTTGGAACGTATCTCC |
| A240M-R | GAGTCGAAACATAGAGAGGATGGGGGCAGC |
| D183N-F | GGACCCCAACTTCCACAAGGGCAACCCGG |
| D183N-R | CCTTGTGGAAGTTGGGGTCCTTGCCAACCTCAC |
| F126H-F | CACAGGGTTACACATCACCACCCACGACGCC |
| F126H-R | GGGTGGTGATGTGTAACCCTGTGTAGAGAAACTCCAGGAC |
| F184Y-F | GACCCCAACTACCACAAGGGCAACCCGGG |
| F184Y-R | GCCCTTGTGGTAGTTGGGGTCCTTGCCAACC |
| F198H-F | GTTTGCTTCTCACATGAGCTCTTACATGTCTCTGTGGC |
| F198H-R | GAGCTCATGTGAGAAGCAAACCAAGGGACCAGAC |
| F244G-F | GTTTCGACTCGGATACTTTGGAACGTATCTCCCCCATAAG |
| F244G-R | GTTCCAAAGTATCCGAGTCGAAACATAGAGAGGATGGGGG |
| H171K-F | CACTGGGAAAAACACAACCACACCGGTGAGG |
| H171K-R | GTGGTTGTGTTTTTCCCAGTGTTTTCTGTGGAGC |
| H185L-F | CCAACTACCTGAAGGGCAACCCGGGTCTG |
| H185L-R | GTTGCCCTTCAGGTAGTTGGGGTCCTTGCCAACC |
| P182Y-F | GGCAAGGACTATAACTACCTGAAGGGCAACCCG |
| P182Y-R | CAGGTAGTTATAGTCCTTGCCAACCTCACCG |
| S197Y-F | GGTTTGCTTACCACATGAGCTCTTACATGTCTCTGTGG |
| S197Y-R | GCTCATGTGGTAAGCAAACCAAGGGACCAGACC |
| W194A-F | CTGGTCCCTGCTTTTGCTTACCACATGAGCTCTTACATGTC |
| W194A-R | GTAAGCAAAAGCAGGGACCAGACCCGGGTTG |
| W194G-F | CTGGTCCCTGGCTTTGCTTACCACATGAGCTCTTACATGTC |
| W194G-R | GTAAGCAAAGCCAGGGACCAGACCCGGGTTG |
| W194L-F | CTGGTCCCTCTGTTTGCTTACCACATGAGCTCTTACATGTC |
| W194L-R | GGTAAGCAAACAGAGGGACCAGACCCGGGTTG |
| W194P-F | CTGGTCCCTCCCTTTGCTTACCACATGAGCTCTTACATG |
| W194P-R | GGTAAGCAAAGGGAGGGACCAGACCCGGGTTG |
| W194R-F | CTGGTCCCTCGATTTGCTTACCACATGAGCTCTTACATG |
| W194R-R | GGTAAGCAAATCGAGGGACCAGACCCGGGTTG |
| W194S-F | CTGGTCCCTTCTTTTGCTTACCACATGAGCTCTTACATGTC |
| W194S-R | GTAAGCAAAAGAAGGGACCAGACCCGGGTTG |

**Table S4. Strains used in this study**

| Strains | Description | Sources |
| --- | --- | --- |
| *E. coli* JM109 | Cloning strain | Lab stock |
| Po1f | ATCC MYA-2613, *ura3-302*, *leu2-270*, *xpr2-322*, *axp-2*, *leu2-270* | Lab stock |
| Ku70 | Po1f, ΔKu70 | Lab stock |
| YL01 | Ku70, E2::*P_TEF_-CarRP^Y27R^-T_XPR2_-P_TEF_-CarB-T_XPR2_* | This study |
| YL02 | YL01, A3::*P_MNDH2_-tHMG1-T_XPR2_- P_TEF_ -GGPPSa-T_XPR2_* | This study |
| YL03 | YL01, A3::*P_MNDH2_-tHMG1-T_XPR2_- P_TEF_ -GGPPXd-T_XPR2_* |  |
| YL04 | YL01, A3::*P_MNDH2_-tHMG1-T_XPR2_- P_TEF_ -GGPPYl-T_XPR2_* |  |
| YL05 | YL01,F1-3::*P_TDH_-MVAE-T_ICLt_- P_MNDH2_ -MVAS- T_XPR2_* | This study |
| YL06 | YL01, E3::*P_TEF_-IDI-T_XPR2_- P_TDH_ –ERG20_MT_^F88S^- T_ICLt_* | This study |
| YL07 | YL01, B1::*P_TEF_-CarRP^Y27R^-T_XPR2_-P_TEF_-CarB-T_XPR2_* | This study |
| YL08 | YL01, E2::*P_TEF_-CarRP^Y27R^-T_XPR2_-P_TEF_-CarB-T_XPR2_*  A3::*P_MNDH2_-tHMG1-T_XPR2_- P_TEF_ -GGPPSa-T_XPR2_*  F1-3::*P_TDH_-MVAE-T_ICLt_- P_MNDH2_ -MVAS- T_XPR2_*  E3::*P_TEF_-IDI-T_XPR2_- P_TDH_ –ERG20_MT_^F88S^- T_ICLt_*  B1::*P_TEF_-CarRP^Y27R^-T_XPR2_-P_TEF_-CarB-T_XPR2_* | This study |
| YL09 | YL08, E13:: *P_TEF_-CarRP^Y27R^-T_XPR2_-P_TEF_-CarB-T_XPR2_* | This study |
| YL10 | YL08, SQS1-50bp | This study |
| YL11 | YL10, A2:: *P_MNDH2_-tHMG1-T_XPR2_- P_TEF_ -GGPPSa-T_XPR2_* | This study |
| YL12 | YL11, B3:: *P_TEF_-Erg12-T_XPR2_-P_TEF_-IDI-T_XPR2_* | This study |
| YL13 | YL12, F4:: *P_TEF_-crtYB-T_XPR2_* | This study |
| YL14 | YL13, E5:: *P_TEF_-ACC1-T_XPR2_* | This study |
| YL15 | YL13, E5:: *P_TEF_-DGA1-T_XPR2_* | This study |
| YL16 | YL15, A1:: *P_TEF_-ZWF1-T_XPR2_-P_TEF_-GND1-T_XPR2_* | This study |
| YL17 | YL16, F17:: *P_TEF_-GND1-T_XPR2_* | This study |
| YL18 | YL17, F1::*P_TEF_-CarRP^Y27R^*-RIAD*-T_XPR2_-P_TDH_–*ERG20_MT_^F88S^*-* RIDD-*T_ICLt_* | This study |
| YL19 | YL17, F1::*P_TEF_-CarRP^Y27R^*-RIDD*-T_XPR2_-P_TDH_–*ERG20_MT_^F88S^*-* RIAD-*T_ICLt_* | This study |
| YL20 | YL17, AXP:: *P_TEF_-IDI*-RIAD*-T_XPR2_-P_TDH_–*GGPPSa*-* RIDD-*T_ICLt_* | This study |
| YL21 | YL20, Δmhy1 | This study |
| Z01-Z18 | YL01-YL18, D17::*P_TEF_ -HpcrtZ-T_XPR2-_P_TDH_-HpBKT-T_ICLt_* | This study |
| Z19 | YL15, D17::*P_TEF_ -HpcrtZ-*RIAD*-T_XPR2-_P-HpBKT-*RIDD*-T_ICLt_* | This study |
| Z20 | Z19, rDNA::*P_TEF_ -HpcrtZ-_TDH_T_XPR2_* | This study |
| Z21 | Z19, rDNA:: *P_TDH_-HpBKT-T_ICLt_* | This study |
| Z22 | Z20, rDNA::*P_TEF_-HpcrtZ^N183A^-*RIAD*-T_XPR2-_P_TDH_-HpBKT^V264D^* -RIDD*-T_ICLt_* | This study |
| Z23 | Z22, rDNA::*P_TEF_-HpcrtZ^N183A^-*RIAD*-T_XPR2-_P_TDH_-HpBKT^V264D^* -RIDD*-T_ICLt_* | This study |
| Z24 | Z23, rDNA::*P_TEF_-HpcrtZ^N183A^-*RIAD*-T_XPR2-_P_TDH_-HpBKT^V264D^* -RIDD*-T_ICLt_* | This study |
| Z25 | Z24, rDNA::*P_TEF_-HpcrtZ^N183A^-*RIAD*-T_XPR2-_P_TDH_-HpBKT^V264D^* -RIDD-KDEL*-T_ICLt_* | This study |
| Z26 | Z24, rDNA::*P_TEF_-HpcrtZ^N183A^-*RIAD*-T_XPR2-_P_TDH_-HpBKT^V264D^* -RIDD-Oleosin*-T_ICLt_* | This study |
| Z27 | Z24, rDNA::*P_TEF_-HpcrtZ^N183A^-*RIAD*-T_XPR2-_P_TDH_-HpBKT^V264D^* -RIDD*-*SKL*-T_ICLt_* | This study |

References

[1] S. A. Haddad, C. C. Lindegren. A method for determining the weight of an individual yeast cell. *Appl. Microbiol.* , 1 (1953) 153-156, <https://doi.org/10.1128/am.1.3.153-156.1953>.
